# Pleiotropic Effects of Sox2 during the Development of the Zebrafish Epithalamus

**DOI:** 10.1371/journal.pone.0087546

**Published:** 2014-01-31

**Authors:** Sofia Pavlou, Katy Astell, Ioannis Kasioulis, Milica Gakovic, Richard Baldock, Veronica van Heyningen, Pedro Coutinho

**Affiliations:** Biomedical Systems Analysis Section, Medical Developmental Genetics Section, Medical Research Council Human Genetics Unit, Medical Research Council Institute of Genetics and Molecular Medicine, University of Edinburgh, Edinburgh, United Kingdom; University of Notre Dame, United States of America

## Abstract

The zebrafish epithalamus is part of the diencephalon and encompasses three major components: the pineal, the parapineal and the habenular nuclei. Using *sox2* knockdown, we show here that this key transcriptional regulator has pleiotropic effects during the development of these structures. Sox2 negatively regulates pineal neurogenesis. Also, Sox2 is identified as the unknown factor responsible for pineal photoreceptor prepatterning and performs this function independently of the BMP signaling. The correct levels of *sox2* are critical for the functionally important asymmetrical positioning of the parapineal organ and for the migration of parapineal cells as a coherent structure. Deviations from this strict control result in defects associated with abnormal habenular laterality, which we have documented and quantified in *sox2* morphants.

## Introduction

Transcriptional networks frequently control finely-tuned processes, such as the development of the central nervous system. SOX2 (sex determining region Y-box 2) is a key transcription factor, which fulfils a multitude of different roles, ranging from acting as a major pluripotency factor required for maintenance of stem cells to proliferation and differentiation in early neurogenesis [1]. Additionally, it fulfils functions in sensory epithelia and some endoderm tissues towards broader organogenesis. Patients with heterozygous loss-of-function mutations in *SOX2* exhibit anophthalmia or microphthalmia [2] and some cases have additional neural phenotypes including learning difficulties, specific motor and brain abnormalities, seizures and anterior pituitary hypoplasia [3]–[7]. The neurological features suggest a role for SOX2 in brain development.

The epithalamus is found in the dorsal diencephalon and consists of three main structures: the pineal gland (epiphysis), the parapineal organ (found only in some species) and the habenular nuclei. The epithalamic structures have been associated with a wide range of biological functions, including circadian rhythms and aspects of sleep, spatial learning and attention, antioxidant protection, stress/fear/anxiety responses, as well as psychotic disorders such as schizophrenia and depression [8]–[10].

The zebrafish pineal gland is a simple structure with only two neuronal cell types: the photoreceptors (PhRs) and the projection neurons (PNs), which derive from the same precursors [11]. These precursors express the homeodomain-containing transcription factor *floating head* (*flh*) and are specified by the Wnt and bone morphogenetic protein (BMP) signaling pathways [11]–[13]. *flh* modulates pineal neurogenesis by controlling the expression of the proneural genes: *achaete-scute complex-like 1a* (*ascl1a)* and *neurogenin 1* (*neurog1)* [11], [14]. Disruption of *flh*, *ascl1a* or *neurog1* leads to reduction in the number of both the PhRs and the PNs [11], [14]. Thus, although these genes are required for neurogenesis, they have no role in cell-fate determination. In contrast, the Notch and BMP pathways have dual roles: they control the total number of pineal cells and inhibit the PN [15] and induce the PhR fate [16], respectively. Understanding the differentiation of this relatively simple pineal gland system will provide insights into the more complex processes of brain development.

The zebrafish epithalamus is also of interest because of its asymmetric architecture. The parapineal is a left-sided organ that specifically innervates the left habenula [17], [18]. At about 30 hours post fertilization (hpf), parapineal cells form a coherent structure at the midline of the anterior pineal anlage and start migrating leftwards. Fgf8a is important for the initiation [19], whereas Nodal signaling controls the laterality of the parapineal migration [17], [18], [20]. In addition, *tbx2b* activity is required for the correct specification of parapineal cells. In the absence of *tbx2b*, fewer cells are specified and these cannot migrate towards the left [21]. Asymmetries in size, cytoarchitecture, connectivity and gene expression are also observed between the left and right habenulae [17], [18], [22]–[24]. Mammalian, including human, brain asymmetries are thought to play a role in behavioral and cognitive functions and their disruption has been associated with disorders, such as schizophrenia, Alzheimer’s disease and learning disabilities [25]. Despite the potential medical relevance, our understanding of the molecular mechanisms underlying these brain asymmetries is still limited.

In this study, we identified and characterized the pleiotropic effects mediated by Sox2 during the development of the epithalamus. We found that Sox2 inhibits pineal neurogenesis and PhR cell fate. We also demonstrated that Sox2 functions independently of the Notch and the BMP pathways during cell-fate determination. Finally, *sox2* knockdown disrupts the asymmetric architecture of the epithalamus. The fact that multiple genes/pathways are involved in the control of these processes highlights the importance of a finely-tuned regulation during brain development.

## Results

### Characterization and Validation of the Knockdown Effect of the *sox2* Morpholino Strategy

To dissect the roles of Sox2 during brain development, particularly in the epithalamus, we established a zebrafish animal model. Since no *sox2* mutant zebrafish lines are available, we designed and tested two different morpholino oligonucleotides (*sox2*-MO1 and *sox2*-MO2), directed against the 5′ sequence near the translation start site (*sox2* is a single-exon gene). Both morpholinos resulted in a fully penetrant and similar phenotype, although of different severities (**Figure S1**).

**Figure 1:**
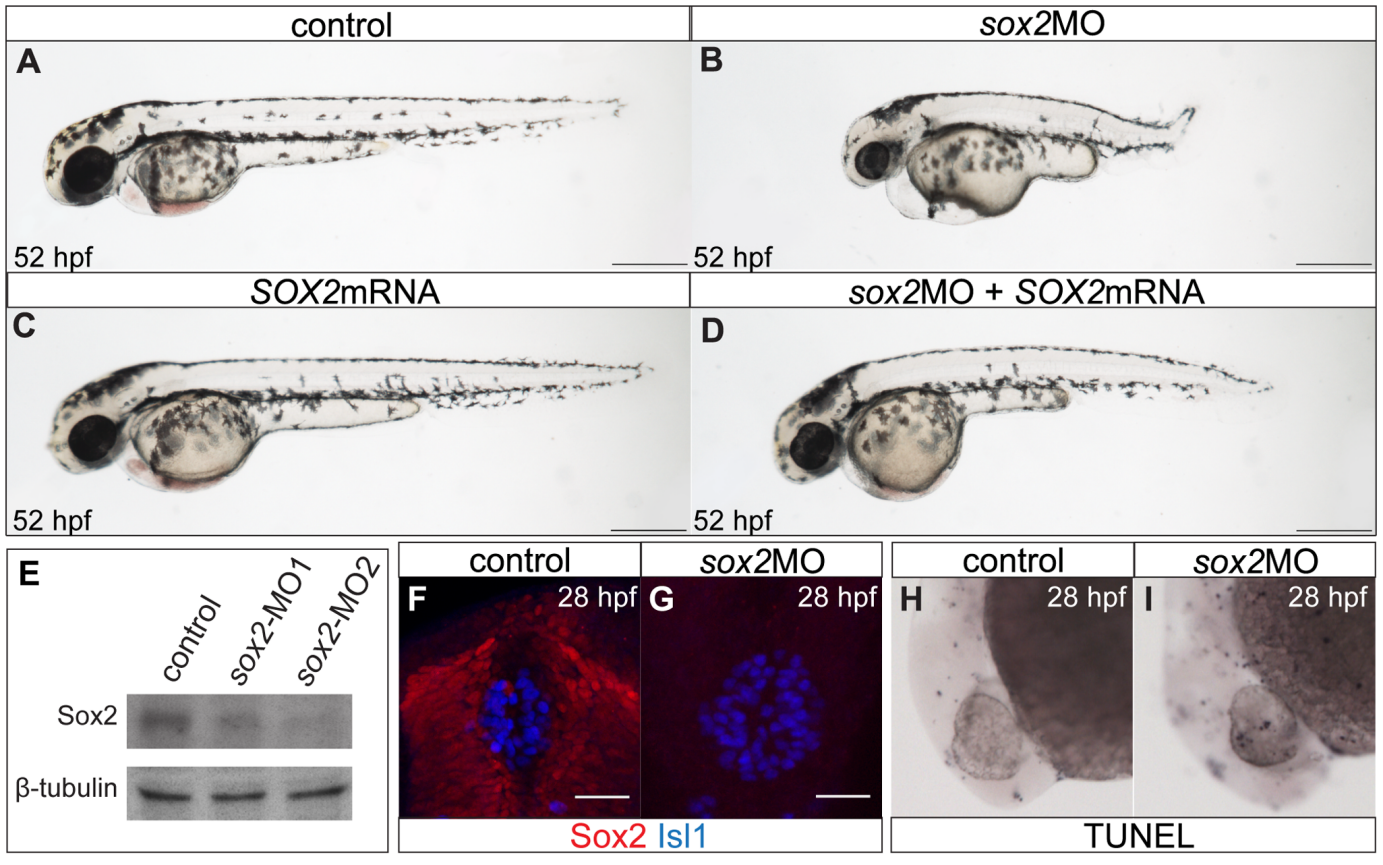
The zebrafish *sox2* morphant model. (**A**) Lateral view of control embryo at 52 hpf. (**B**) *sox2* morphants have small eyes and short body axis in relation to control siblings. (**C**) Microinjections with *SOX2* mRNA have no phenotypic effect, while (**D**) mRNA injections into *sox2* morphants rescue the phenotypes. (**E**) Western blot showing the reduction in the levels of Sox2 in embryos injected with *sox2*-MO1 (second column) and *sox2*-MO2 (third column), when compared to controls (first column). β-tubulin was used as loading control. (**F**) Whole mount immunofluorescence for Sox2 (red) and Isl1 (blue) shows that Sox2 is expressed in the epithalamus, but not in the differentiated pineal cells at 28 hpf. (**G**) Sox2 is undetectable by immunofluorescence in *sox2* morphants, at 28 hpf. (**H–I**) Morpholino microinjections do not grately affect apoptosis, as judged by TUNEL assay at 28 hpf. (**A–D**) scale bars = 250 µm, (**F–G**) scale bars = 25 µm. See also **Figure S1**.

*sox2* morphants have a shorter anteroposterior axis and smaller eyes in relation to control siblings (**Figure 1A–B**). This mirrors the most common phenotype observed in patients with *SOX2* mutations [2]. Also, knockdown of *sox2* results in early lethality: morphants die at around 5 dpf. Western blot analysis, using whole-embryo protein extracts at 28 hpf, shows that either morpholino (at a final concentration of 3.5 ng) results in decreased levels of Sox2 protein (**Figure 1E**). Densitometric analysis of the western blot revealed that *sox2*-MO1 results in approximately 66% reduction, where as *sox2*-MO2 results in 80% reduction. Since *sox2*-MO2 is the most efficient, it was used throughout this study. Whole mount immunofluorescence against Sox2, in control and *sox2* morphant siblings, further confirms the efficiency of the knockdown (**Figure 1F–G**).

Interestingly, the severity of the phenotype correlates with the levels of *sox2* knockdown, as well as the specific concentration of the different morpholinos injected (**Figure S1**). At the dose used for our experiments, the number of apoptotic cells is similar between control and morphant siblings, at 28 hpf (**Figure 1H–I**). We do not observe discoloured tissues around the eyes and the nervous system of live embryos, a characteristic of off-targeting effects [26]. Furthermore, to show the specificity of the phenotype, we rescued the morpholino-induced defects by co-injecting human *SOX2* mRNA (**Figure 1D**). Notably, overexpression of the human *SOX2* in zebrafish embryos does not result in any phenotypic effect (**Figure 1C**).

### Sox2 is Expressed in the Pineal Precursors and Downregulated in Differentiated Pineal Cells

**Figure 2:**
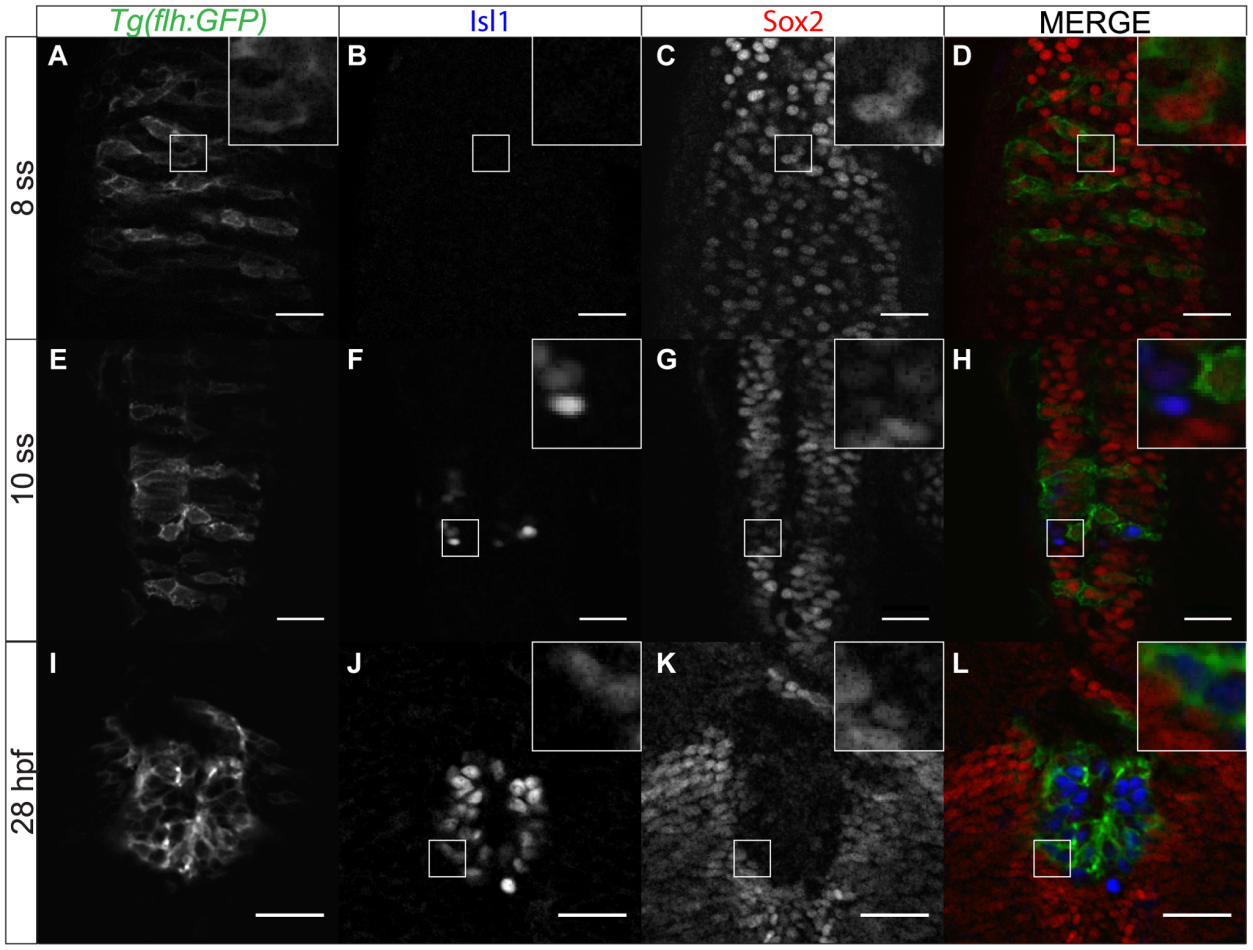
Sox2 expression within the pineal anlage is downregulated with differentiation. (**A–D**) Sox2 expression overlaps with expression of *flh*, a marker for pineal precursors, at 8 ss. (**E–H**) As pineal cells start differentiating, Isl1 is upregulated whereas Sox2 is downregulated. Yellow arrows show cells that have both Isl1 and Sox2 expression at low levels. (**I–L**) Sox2 is absent from the fully differentiated pineal cells. Scale bars = 25 µm, optical sections from confocal microscopy, insets show a three times magnified view of the image, (**E,I,M**) GFP expression of *Tg(flh:GFP)* (**F,J,N**) immunofluorescence for Isl1, (**G,K,O**) immunofluorescence for Sox2, (**H,L,P**) merged images with *Tg(GFP:flh)* in green, Isl1 in blue and Sox2 in red, developmental stages are shown at the beginning of each row.

In order to dissect the roles of Sox2 in the developing epithalamus, we first examined its expression profile. At approximately 8 somite stage (ss), Sox2 is expressed throughout the developing epithalamus, including the presumptive pineal gland, which is GFP-positive in *Tg(flh:GFP)* embryos (**Figure 2A–D**). From about 10 ss, some cells start expressing Isl1, a marker for differentiated pineal neurons. Sox2 expression is downregulated in these Isl1-positive cells and by 28 hpf, Sox2 is completely excluded from the fully differentiated pineal gland (**Figure 2E–L**). Thus, Sox2 is expressed in pineal precursors and its expression is downregulated following neurogenesis. This is in agreement with previous studies, which suggest that Sox2 is generally expressed in neuronal progenitor cells and its expression is downregulated with differentiation [27], [28].

### Sox2 Inhibits Neurogenesis within the Pineal Gland

**Figure 3:**
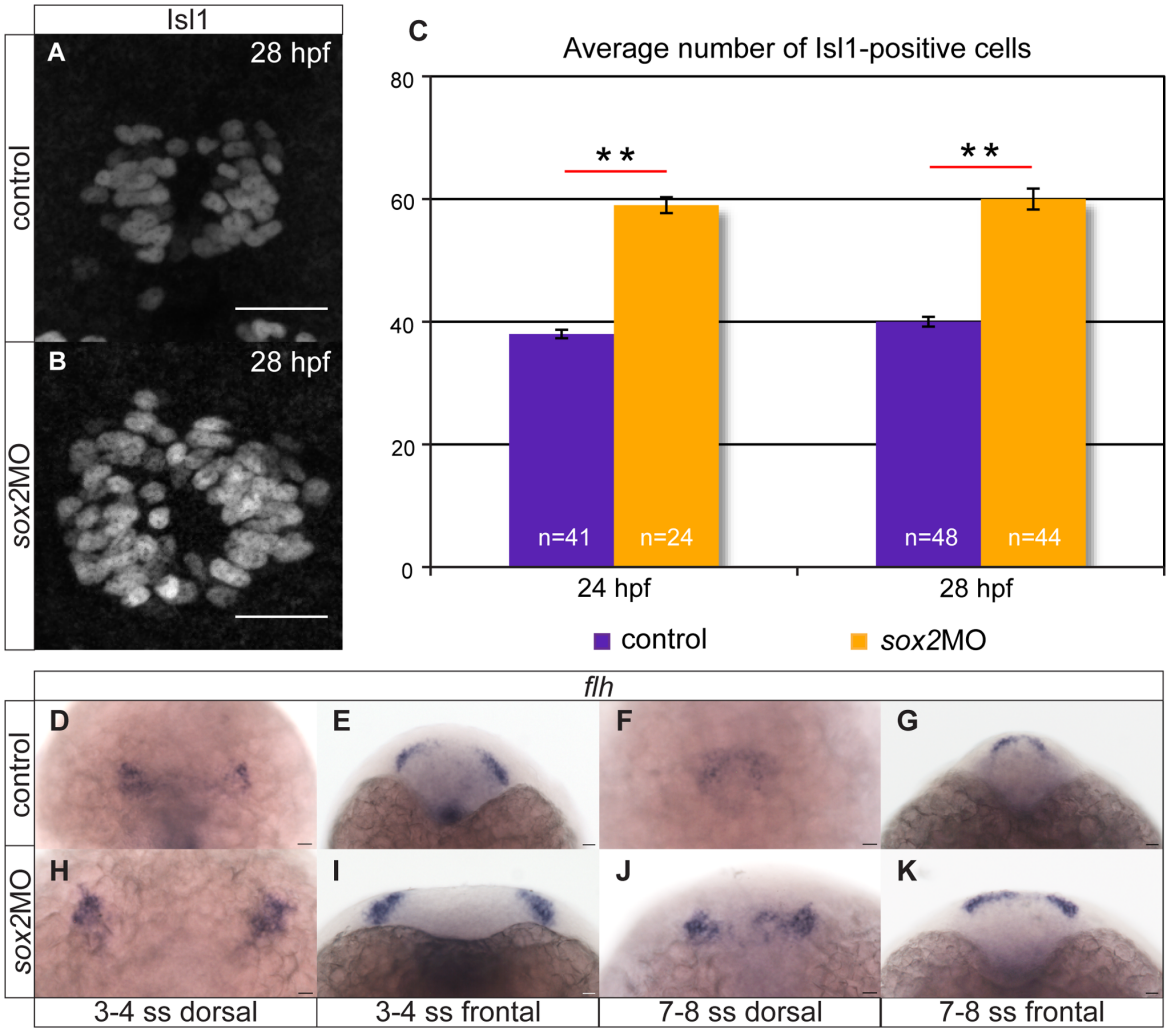
Knockdown of *sox2* results in upregulation of neurogenesis. (**A–B**) Neurogenesis is increased in *sox2* morphants, as judged by Isl1 expression. (**C**) Average number of Isl1-positive cells in control (purple bar) and *sox2* morphants (orange bar), at 24 and 28 hpf. (**D–E**) At 3–4 ss, two *flh*-domains are observed on either side of the neural plate. (**F–G**) By 7–8 ss, the two domains fuse to form the presumptive pineal gland. (**H–K**) In *sox2* morphants, *flh* is expressed in broader domains in relation to control siblings. Scale bars = 25 µm, (**A–B**) confocal maximum projections, (**C**) error bars represent ± standard error, ** = p-value <0.001 (MWU test). (**J,L,N,P**) dorsal views with anterior to the top, (**K,M,O,Q**) frontal views. See also **Figure S2–S3**.

It is known that the correct levels of Sox2 are vital for the maintenance of neuronal progenitor identity and for cell-cycle exit and differentiation of progenitor cells [27]. We, therefore, investigated pineal neurogenesis in *sox2* morphants. We performed whole mount *in situ* hybridizations (**Figure S2**) and immunofluorescence for Isl1 (**Figure 3A–C**), a protein expressed in all pineal neurons (i.e. both PhRs and PNs) [11]. Knockdown of *sox2* results in approximately 50% increase in the number of Isl1-positive cells: control embryos have on average 38 Isl1 cells at 24 hpf and 40 Isl1 cells at 28 hpf, whereas *sox2* morphants have 59 and 60 cells, respectively (Mann-Whitney U test (MWU); p-value <0.001) (**Figure 3A–C**). Notably, the number of Isl1-positive cells is not significantly affected in embryos injected with control morpholinos (43 Isl1 cells at 28 hpf), when compared with uninjected siblings (40 Isl1 cells at 28 hpf, MWU; p-value = 0.19). These data suggest that Sox2 inhibits neurogenesis within the developing pineal gland.

To determine whether the increase in neurogenesis observed in *sox2* morphants is due to an increased pool of precursor cells, we assayed *flh* expression. *flh* is first detected at approximately 3–4 ss in two domains on each side of the neural plate. As neurulation proceeds, these *flh*-positive domains fuse to form the presumptive pineal gland [11]. In *sox2* morphants, *flh* is expressed in a broader domain at early stages (3–8 ss) (**Figure 3D–K**), but becomes normal at later stages (16–32 hpf) (**Figure S3A–H** and data not shown). The expanded territory of *flh* observed in *sox2* morphants may contribute to the increased neurogenesis. The fact that *flh* expression is restored at later stages suggests that a second Sox2-independent mechanism is also involved in the regulation of *flh*. Notably, the fusion of the two *flh*-positive domains on each side of the neural tube is delayed in *sox2* morphants (compare **Figure 3F** with **3J**). However, at 24 hpf, a single *flh* domain is observed (**Figure S3A–H**), demonstrating that the closure of the neural tube is not affected.

*flh* regulates neurogenesis by modulating the proneural genes *ascl1a* and *neurog1* [11], [14]. Disruption of *ascl1a* leads to reduction in the number of pineal cells and simultaneous knockdown of *ascl1a* and *neurog1* results in a more severe phenotype. In contrast, knockdown of *neurog1* alone does not cause any phenotype. Since *flh* expression is abnormal in *sox2* morphants, we decided to investigate whether *ascl1a*, the proneural gene with the major effect, is also affected. We found that *ascl1a* expression is increased at 16 hpf, which can explain the increased neurogenesis observed when *sox2* is knocked down (**Figure S3M–T**).

### Sox2 Controls Cell-fate Determination within the Pineal Gland

Since neurogenesis is upregulated in *sox2* morphants, we examined the identity of the extra neurons generated. The pineal gland consists of two cell types: PhRs, which express *aanat2* (*arylalkylamine N-acetyltransferase*) and PNs that express *pax6* and *elavl3* (*ELAV (embryonic lethal, abnormal vision, Drosophila)-like 3 (Hu antigen C)*) [11], [15], [29].

**Figure 4:**
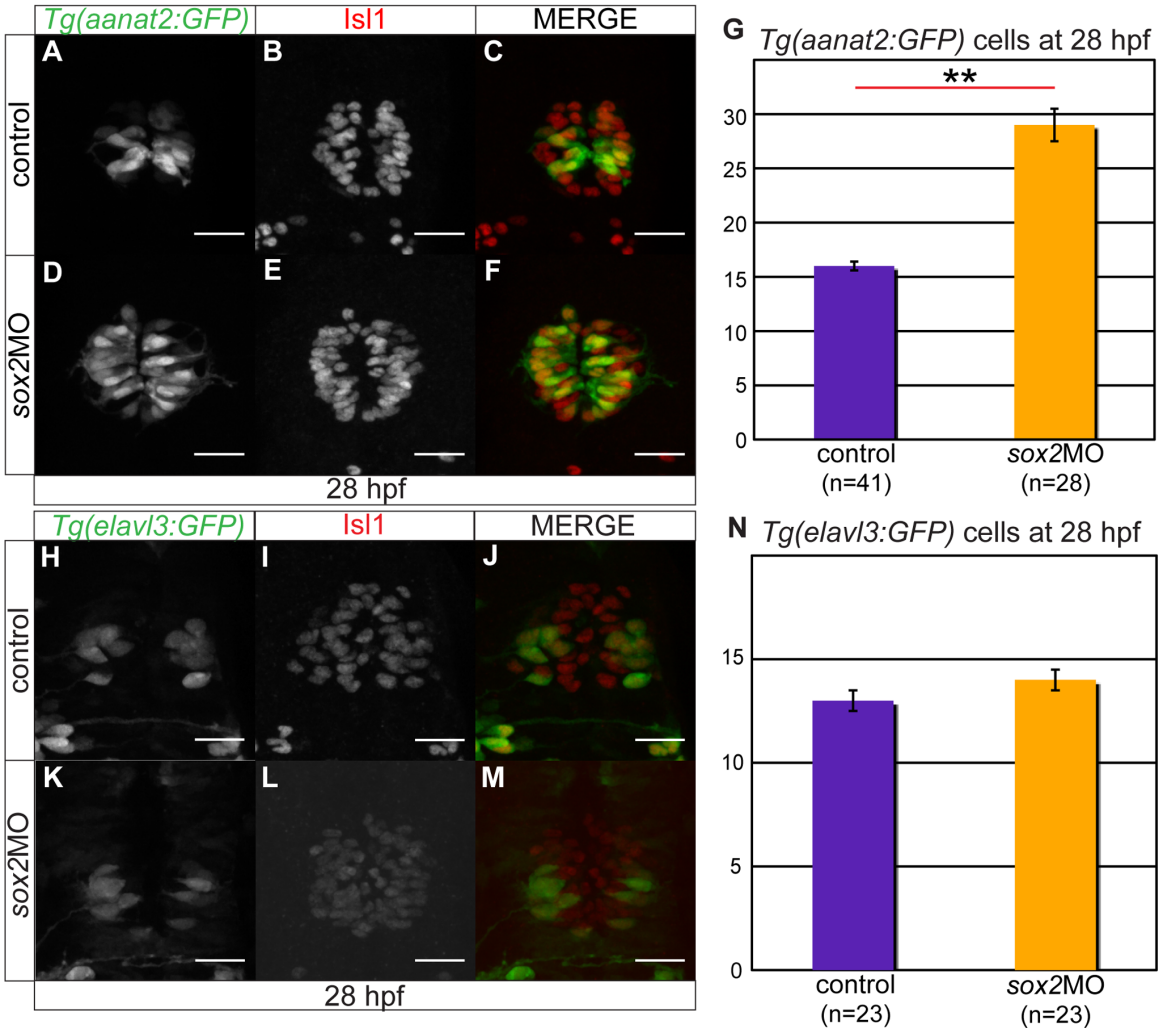
Sox2 inhibits the PhR cell fate. (**A–C**) *Tg(aanat2:GFP)* drives GFP expression in the pineal PhRs. (**B**) Isl1 labels the pineal cells. (**D–F**) Knockdown of *sox2* results in increased number of PhRs. (**G**) The average number of PhRs in control (purple bar) and *sox2* morphants (orange bar). (**H–J**) *Tg(elavl3:GFP)* drives GFP expression specifically in the PNs. (**K–M**) The knockdown of *sox2* does not affect the number of PNs. (**N**) The average number of PNs in controls (purple bar) and *sox2* morphants (orange bar) does not significantly differ. Confocal maximum projections of 28 hpf embryos, scale bars = 25 µm, error bars represent ± standard error, ** = p-value <0.001 (MWU test). See also **Figure S4**.

The number of PhRs is highly increased in *sox2* morphant embryos, as shown by whole mount *in situ* hybridizations for *aanat2* (**Figure S4A–H**) and GFP expression in *Tg(aanat2:GFP)* embryos (**Figure 4A–G**). Quantitatively, *sox2* knockdown results in almost two-fold increase in the number of PhRs, at 28 hpf (16 cells in controls and 29 cells in *sox2* morphants, MWU test; p-value <0.001).

To investigate whether the number of PNs is also affected in *sox2* morphants, we performed whole mount *in situ* hybridizations for *pax6b* (**Figure S4I–N**) and analyzed the GFP expression in *Tg(elavl3:GFP)* embryos (**Figure 4H–N**). The number of PNs is not affected in *sox2* morphants, at 28 hpf: control embryos have on average 13 PNs and *sox2* morphants have 14 PNs (MWU test, p-value = 0.058) (**Figure 4N**). In spite of that, later in development (48 hpf), some of the PNs are mis-positioned in *sox2* morphants, as judged by whole mount *in situ* hybridizations, possibly as a result of the increased number of PhRs present in the centre of the pineal gland (**Figure S4K,N**).

Altogether, the data suggest that Sox2 inhibits the PhR fate, but it has no function in controlling PN fate.

### Sox2 and Notch Complement Each other in Cell-fate Determination

Notch signaling is known to inhibit neurogenesis within the pineal gland [15]. Since Sox2 also inhibits neurogenesis, we investigated whether Notch signaling and Sox2 work in the same or different pathway(s). To achieve this, we analyzed whether inhibition of *sox2* affects Notch signaling and *vice versa*.

To assess Notch signaling in *sox2* morphants, we validated (data not shown) and used the Notch-reporter transgenic line *Tg(csl:venus)*. At both 24 and 28 hpf, there is no significant difference in the number of venus-positive cells between *sox2* morphant and control siblings (MWU test; p-value = 1 for both stages) (**Figure S5**). Thus, Sox2 does not function upstream of Notch in modulating neurogenesis within the pineal gland.

We then assessed Sox2 expression in embryos with compromised Notch activity. For this, we used the transgenic line *Tg(flh:GFP)* to visualize the presumptive pineal gland and immunofluorescence for Isl1 to mark the differentiated pineal cells. Knockdown of Notch, achieved via treatment with the inhibitory drug DAPT, does not qualitatively affect Sox2 expression within the pineal gland: Sox2 is still expressed in undifferentiated *flh*-positive cells and is downregulated in differentiated Isl1-expressing cells (**Figure S6** and **Movie S1**). However, inhibition of Notch results in increased number of Isl1 cells: 13 Isl1-positive cells in DMSO-treated controls (n = 7) and 23 Isl1-positive cells in DAPT-treated embryos (n = 4). As a consequence, *sox2* is downregulated in a broader domain in relation to control siblings. In contrast, upregulation of the Notch signaling (via heat-shock of *Tg(hs:Gal4);Tg(UAS:Notch-intra)* double transgenics) results in reduced number of Isl1-positive cells: 20 Isl1-positive cells in controls (n = 6) and 6 Isl1-positive cells in embryos with ectopic Notch signaling (n = 5). Consequently, Sox2 is expressed in a broader domain in these embryos when compared to controls (**Figure S7** and **Movie S2**). To conclude, disruption of Notch signaling affects the number of Sox2-expressing cells within the epithalamus. However, we cannot determine whether the differences in Sox2 expression are a direct effect of the compromised Notch signaling or a secondary effect caused by the aberrant neurogenesis in these animals.

**Figure 5:**
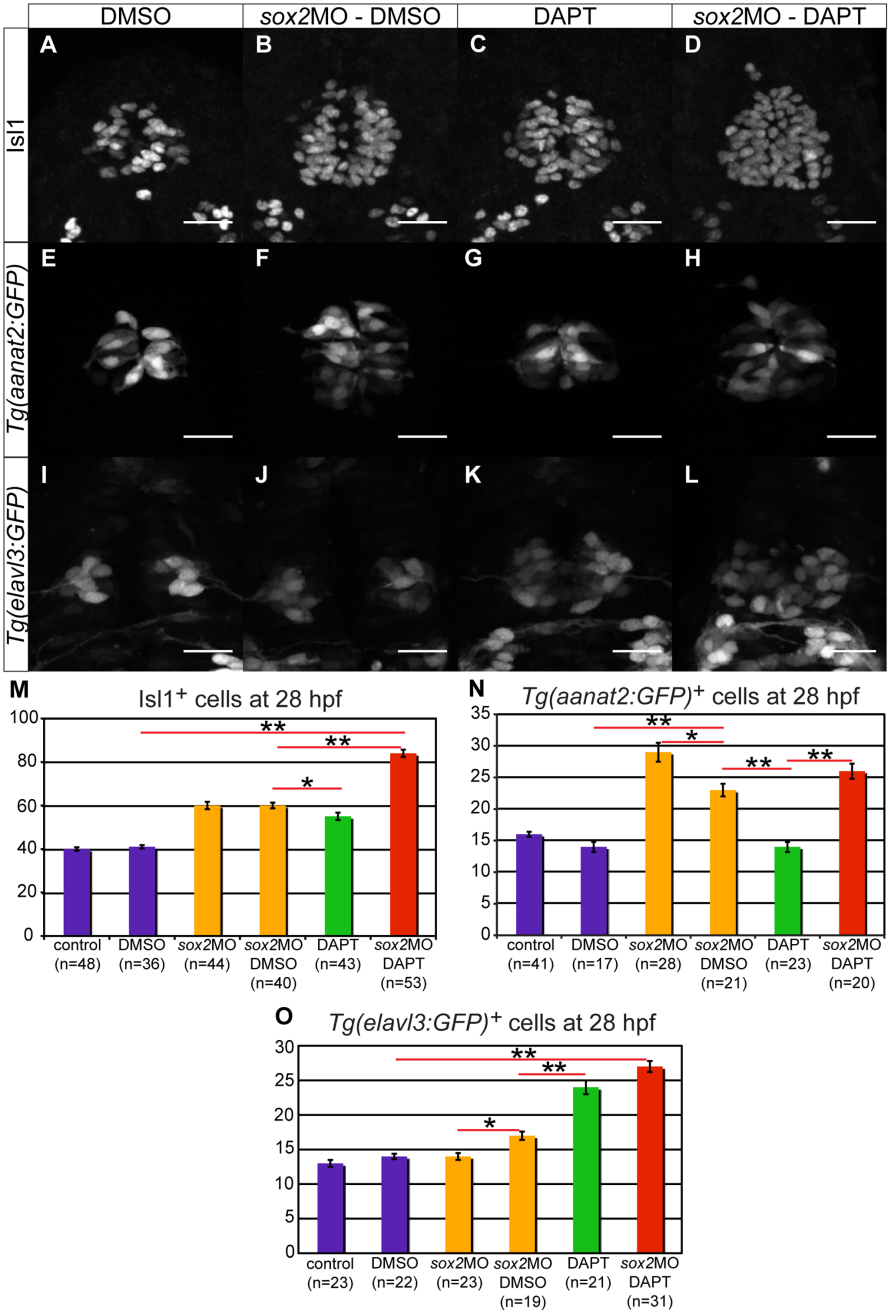
Sox2 and Notch have synergistic effect on neurogenesis and complementary effects on cell-fate determination. (**A–D**) The number of pineal neurons (Isl1-positive cells) is increased in *sox2* morphants (**B**) and in DAPT-treated embryos (**C**), when compared to DMSO-treated controls (**A**). A synergistic effect is observed when both *sox2* and Notch are knocked down (**D**). (**E–H**) *Tg(aanat2:GFP)* drives GFP expression in the pineal PhRs in DMSO-treated controls (**E**). The number of PhRs is increased in *sox2* morphants (**F**), but remains unaffected in DAPT-treated embryos (**G**). The knockdown of both *sox2* and Notch results in an increased number of GFP-positive cells (**H**), comparable to the one observed in *sox2* morphants. (**I–L**) *Tg(elavl3:GFP)* expresses GFP in PNs. There is no difference in the number of GFP-positive in *sox2* morphants (**I**) when compared to DMSO-treated controls (**I**). The knockdown of Notch alone (**K**) or simultaneously with *sox2* (**L**) results in similar increase in GFP expression. (**M–O**) Average number of Isl1-positive cells (**M**), PhRs (**N**) and PNs (**O**) in untreated controls, DMSO-treated controls, untreated *sox2* morphants, DMSO-treated *sox2* morphants, DAPT-treated embryos and DAPT-treated *sox2* morphants. Confocal maximum projections of 28 hpf embryos, scale bars = 25 µm, error bars represent ± standard error, * = p-value <0.05 (MWU test), ** = p-value <0.001 (MWU test). See also **Figure S5–S7** and **Movie S1–S2**.

Since both Sox2 and Notch are important for neurogenesis and cell-fate determination within the pineal gland, we decided to investigate the effects of their simultaneous misregulation. We found that inhibition of both *sox2* and Notch results in a synergistic effect on neurogenesis: the number of Isl1-positive cells observed in embryos with compromised Sox2 and Notch activity is greater than the number expected from the sum of the individual knockdowns (84 Isl1-positive cells instead of the expected 75 cells, chi-square test; p-value <0.001; absolute standardized residual >2) (**Figure 5A–D,M**).

In contrast, knockdown of both *sox2* and Notch results in additive effect on cell-fate determination: the number of PhRs and PNs observed in these embryos is similar to the number expected from the sum of the individual effects (chi-square test; p-value >0.05) (**Figure 5E–L,N–O**).

Although an additive effect is widely accepted to show that two genes/pathways function independently, the interpretation of synergy is controversial [30]. This is mainly due to the difficulties in distinguishing between synergy and additivity. Therefore, some researches consider synergy to be similar to additivity (suggesting an absence of functional relationship), whereas others believe that synergy demonstrates a functional relationship (see discussion) [30]. Here, the number of the extra neurons generated is small (albeit statistically significant), making the distinction between additivity and synergy difficult. Therefore, the data suggest (but do not prove) that Sox2 and Notch pathway function synergistically to modulate pineal gland neurogenesis, whereas they complement each other in cell-fate determination.

### Sox2 Controls PhR Identity Independently of the BMP Pathway

The BMP pathway is also involved in PhR cell-fate determination [16] and thus we decided to investigate the relationship between Sox2 and BMP. We began by assessing the expression profile of Sox2 in embryos with compromised BMP signaling and *vice versa*.

Embryos were treated with different concentrations (30, 40 and 50 µM) of dorsomorphin, a drug known to inhibit BMP signaling [31], at 6 hpf. At this stage, inhibition of BMP led to dorsalization phenotypes (data not shown), confirming the efficiency of the drug. Within the epithalamus, Sox2 expression is unaffected in dorsomorphin-treated embryos (**Figure S8**, **Movie S3** and data not shown), suggesting that BMP does not act upstream Sox2 in modulating PhR cell fate.

In order to investigate whether Sox2 controls BMP activity, we used the BMP-reporter *Tg(BRE:GFP)* [32]. This reporter expresses a destabilized version of GFP, with a shorter half-life as compared to enhanced GFP, and it can therefore be used to study more acute signaling events.

**Figure 6:**
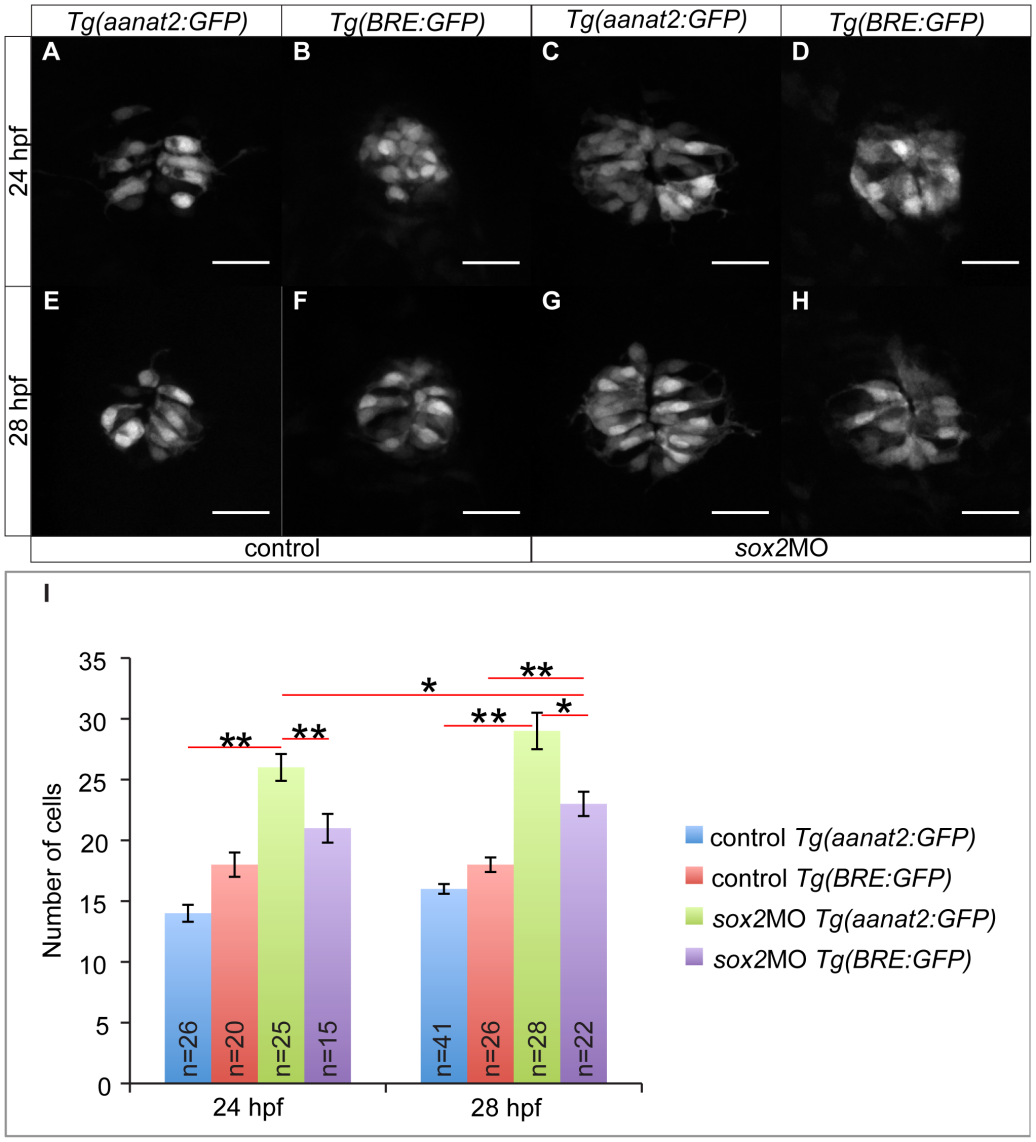
Sox2 controls PhR cell fate independently of BMP. (**A**) The transgenic line *Tg(aanat2:GFP)* marks the PhRs. (**B**) The transgenic line *Tg(BRE:GFP)* is as a BMP signaling reporter. (**C**) In *sox2* morphant embryos, the number of PhRs is increased at 24 hpf, when compared to controls (**A**). (**D**) The number of BMP-responsive cells at 24 hpf is unaffected when *sox2* is knocked down. (**E–F**) The number of PhRs (**E**) and BMP-responsive cells (**F**) is similar in control embryos at 28 hpf. (**G–H**) The number of PhRs (**G**) is higher than the number of BMP-responsive cells (**H**) at 28 hpf, in *sox2* morphants. (**I**) The average number of PhRs (*Tg(aanat2:GFP)*-positive cells) and BMP-responsive cells (*Tg(BRE:GFP)*-positive cells) in control and *sox2* morphant embryos, at 24 and 28 hpf. Confocal maximum projections, scale bars = 25 µm, error bars represent ± standard error, ** = p-value <0.001 (MWU test). See also **Figure S8** and **Movie S3**.

At 24 hpf, BMP activity is normal when *sox2* is knocked down (18 GFP-positive cells in controls and 21-positive cells in *sox2* morphants; MWU test; p-value = 1) (**Figure 6A–B,I**). However, at 28 hpf, there is a significant increase in the number of GFP-positive cells in *sox2* morphants in relation to controls (18-positive cells in controls and 23-positive cells in morphants, MWU test; p-value <0.001) (**Figure 6E–F,I**). Thus, our data suggest that *sox2* knockdown affects BMP activity. To elucidate this further, we checked whether the number of PhRs is affected at 24 hpf, a stage at which BMP signaling is normal in *sox2* morphants. We found that knockdown of *sox2* results in a significantly increased number of PhRs even at 24 hpf (14 PhRs in controls and 26 PhRs in *sox2* morphants, MWU test; p-value <0.001) (**Figure 6**), suggesting that Sox2 controls the PhR fate independently of BMP signaling. In addition, the increase in the number of PhRs observed in *sox2* morphants is higher than the increase in BMP-responsive cells. In particular, *sox2* morphants have on average 12 and 13 extra PhRs at 24 and 28 hpf, respectively, in relation to control siblings. In contrast, *sox2* knockdown results in 3 and 5 extra BMP-responsive cells at 24 and 28 hpf, respectively, when compared to controls. Finally, in *sox2* morphants, the number of *aanat2*-positive cells (PhRs) at 24 hpf is significantly higher than the number of BMP-responsive cells at 28 hpf (MWU test; p-value = 0.031) eliminating the hypothesis that this is an artefact due to delay in the activation of the destabilized GFP.

In order to further test our hypothesis that Sox2 modulates PhR fate independently of the BMP pathway, we treated *sox2* morphants with dorsomorphin and analyzed the number of PhR. If Sox2 inhibits the PhR fate independently of the BMP, then an increased number of PhR will be observed even when BMP is absent. As shown in **Figure S9**, dorsomorphin treatment (40 µM) results in a significant reduction in the number of PhR: 19 PhRs in DMSO-treated controls and 13 PhRs in dorsomorphin-treated embryos (MWU test; p-value = 0.027). This is in agreement with previous studies demonstrating that BMP normally promotes the PhR fate [16]. Contrary, *sox2* morphants treated with dorsomorphin have on average 19 PhRs, which is similar to controls (MWU test; p-value = 1) and significantly increased in relation to dorsomorphin-treated embryos (MWU test; p-value = 0.029). Therefore, knockdown of *sox2* is able to overcome the effects of a compromised BMP pathway. The fact that dorsomorphin-treated *sox2* morphants have fewer PhR is relation to *sox2* morphants treated with DMSO (MWU test; p-value <0.001) demonstrates that BMP is equally important for the proper specification of PhR. Together, the data show that Sox2 and the BMP pathway function via two independent mechanisms, where they inhibit and promote the PhR fate, respectively. Disruption of any of the two leads to abnormal number of PhRs, whereas disruption of both can rescue the phenotype.

### *sox2* Knockdown Disrupts Parapineal and Habenular Development

**Figure 7:**
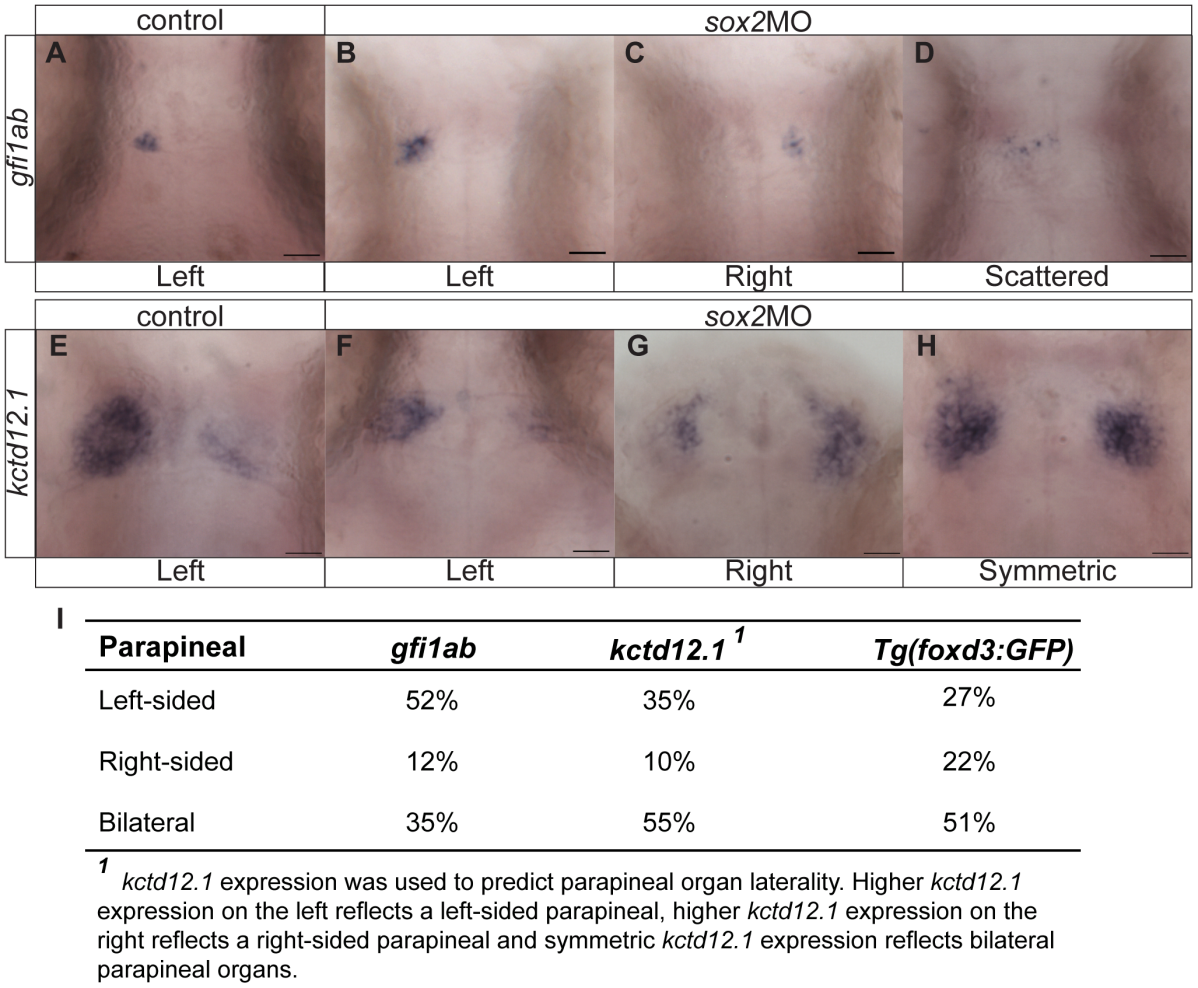
Knockdown of *sox2* results in abnormal parapineal development and disruption of the habenular asymmetries. (**A**) *gfi1ab* is expressed in parapineal cells on the left side of the brain in control embryos. (**B–D**) *sox2* morphants are categorized into three groups according to *gfi1ab* expression: left-sided expression (**B**), right-sided (**C**) and embryos with scattered *gfi1ab* cells (**D**). (**E**) *kctd12.1* is asymmetrically expressed in the habenulae, with a broader expression domain in the left than the right habenula. (**F–H**) In *sox2* morphants *kctd12.1* expression can be: asymmetric with more on the left side similar to control embryos (**F**), asymmetric with more on the right side (**G**) or symmetric (**H**). (**I**) A table showing the percentage of embryos with normal, reversed or bilateral parapineal organs, using different staining methods. Scale bars = 25 µm. See also **Figure S9–S10** and **Movie S4.**

**Figure 8:**
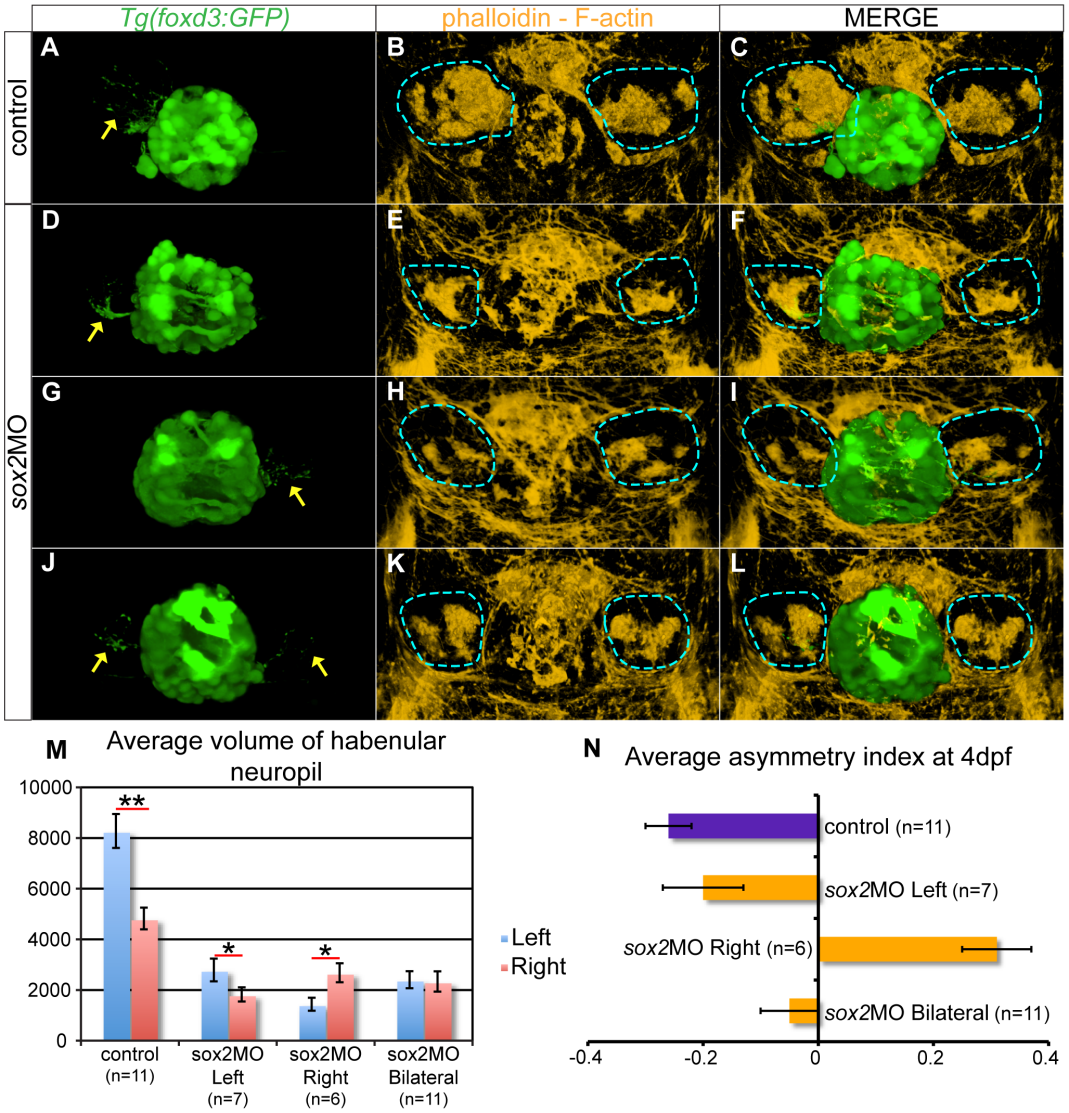
The parapineal and habenular defects are coupled in *sox2* morphants. (**A–C**) In control embryos, the left-sided parapineal projects towards the left habenula. As a result, the left habenula has denser neuropils than the right, as judged by phalloidin staining. (**D–F**) In *sox2* morphants with left-sided parapineal projections, the left habenula is larger than the right. (**G–I**) Morphants with right-sided parapineal organs display reverse habenular asymmetries, whereas (**J–L**) morphants with bilateral parapineal projections have symmetric habenulae. (**M**) The average volume of the left (blue bars) and right (red bars) habenular neuropils, as judged by the volume of phalloidin-positive areas within the habenulae. y-axis show volume in µm^3^. (**N**) Average asymmetry index in control (purple bar) and *sox2* morphants (orange bars). 3D reconstructions of confocal images at 4 dpf, arrows show parapineal projections and blue lines surround the habenular neuropils, error bars represent ± standard error, (**M**) * = p-value <0.05 and ** = p-value <0.001 (Wilcoxon test).

The fact that Sox2 is expressed throughout the zebrafish epithalamus led us to hypothesize that it may also control parapineal and habenular development. To test this, we performed whole mount *in situ* hybridizations for *gfi1ab* (*growth factor independent 1ab*), a gene specifically expressed in parapineal cells [33]. In control embryos, *gfi1ab* was always detected on the left side of the brain. In contrast, *sox2* morphants were categorized into three groups according to their *gfi1ab* expression: embryos with left-sided, right-sided or scattered *gfi1ab*-positive cells (**Figure 7A–D**). These phenotypes were confirmed using the transgenic line *Tg(foxd3:GFP)* that marks both the pineal and parapineal cells (**Figure 8A,D,G,J**). In addition, we observed that embryos with scattered parapineal cells are able to project to both the left and right habenulae and so we refer to these embryos as having bilateral parapineal organs. Depending on the staining method, some variability in the percentage of embryos falling into each category is observed (**Figure 7I**). We suspect that GFP expression of *Tg(foxd3:GFP)* embryos is more representative, since single scattered parapineal cells may not be detected by whole mount *in situ* hybridizations.

We performed timelapse experiments using *Tg(foxd3:GFP)* embryos to observe parapineal migration in live embryos. In control embryos, parapineal cells migrate towards the left, as previously described [21]. However, in *sox2* morphants, parapineal cells either migrate towards the left or migrate towards the right or are misguided and disconnected from the group resulting in scattered (bilateral) parapineal organs (**Movie S4**).

Previous studies suggest that the presence of a left-sided parapineal organ is essential for the establishment of habenular asymmetries [22]. Since the parapineal is abnormal in *sox2* morphants, we hypothesized that the habenulae may also be affected. To investigate this, we performed whole mount *in situ* hybridizations for *kctd12.1* (*potassium channel tetramerisation domain containing 12.1*) in control and *sox2* morphant siblings. *kctd12.1* is predominantly expressed in the left habenula with fewer positive cells in the right habenula (**Figure 7E**) [22]. However, *sox2* morphants can be categorized into three groups based on their *kctd12.1* expression: embryos with normal-like, reversed or symmetric (left isomerism) expression (**Figure 7F–I**). Notably, in embryos with normal-like *kctd12.1* expression, the positive domain in the left habenula is larger than the right, but is reduced when compared to control embryos.

Since *sox2* morphants display abnormalities in both the parapineal and habenulae, we hypothesized that the two defects may be coupled. To confirm this, we stained *Tg(foxd3:GFP)* embryos with phalloidin, a marker for filamentous actin (F-actin), which is enriched in neuropil areas [34], [35]. The zebrafish habenulae consist of neuropil-rich areas in the centre surrounded by neuron bodies [36], [37]. In addition, the left neuropils are larger than right.

*Tg(foxd3:GFP)* embryos labeled with phalloidin were imaged and 3D reconstructions were generated. We measured the volume of the left and right phalloidin-positive neuropil areas for each embryo and calculated the asymmetry index (AI) as previously described [38]. By definition, AI values between −1 and 0 correspond to a larger left-sided neuropil and values between 0 and 1 indicate a larger right neuropil. In control embryos, the left habenular neuropil is larger than the right (Wilcoxon paired signed rank test (Wilcoxon); p-value <0.001), with an AI of −0.26 (**Figure 8**). For the *sox2* morphants, we first assigned the embryos according to the position of the parapineal organ (left, right and bilateral) based on the GFP-expression of *Tg(foxd3:GFP)* embryos. We found that the habenula that receives parapineal projections is always the largest in *sox2* morphants, even though the difference between the two habenulae is smaller in relation to controls (**Figure 8**). In particular, embryos with left-sided parapineal organs have enlarged left habenula (Wilcoxon; p-value <0.05, AI = −0.2), whereas embryos with right-sided parapineal organs have a larger right habenula (Wilcoxon; p-value <0.05, AI = 0.31). Finally, *sox2* morphants with bilateral parapineal organs have symmetric habenular neuropils (Wilcoxon; p-value >0.05, AI = −0.05).

In conclusion, Sox2 is required for the proper development and migration of the parapineal cells and as a consequence for the proper establishment of habenular asymmetries.

### Abnormal Bilateral Activation of Nodal Pathway Accounts for the Reverse-parapineal Phenotype Observed in *sox2* Morphants

The *sox2* morphant parapineal phenotypes led us to investigate whether *tbx2b*, *fgf8a* or Nodal activity are affected in these embryos.

We found that *tbx2b* is downregulated in *sox2* morphants in relation to control siblings, at all stages analyzed (**Figure S10**). Since the number of *gfi1ab*-positive cells appears to be normal in *sox2* morphants, we hypothesize that the reduced *tbx2b* expression results in loss of some parapineal-cell properties that are necessary for collective rather than individual migration of cells. Therefore, the downregulation of *tbx2b* may partly account for the scattered parapineal cells observed in *sox2* morphants.

The growth factor *fgf8a* is bilaterally expressed in the epithalamus and during parapineal migration its expression is higher on the left than the right side [19] (**Figure S11**). *sox2* morphants have normal *fgf8a* expression. However, as development proceeds, *fgf8a* expression is restricted to the medial part of the diencephalon in control embryos, while in *sox2* morphants *fgf8a* is expressed in a broader domain (**Figure S11C–D’**). Since Fgf8a functions as a chemoattractant, this broader *fgf8a* expression may be an additional factor contributing to the scattering effect observed in *sox2* morphants.

In contrast to the *fgf8a*, the Nodal pathway is transiently activated only on the left side of the brain, between 18 and 28 hpf. Previous studies demonstrated that absent or bilateral activation of Nodal leads to randomization of parapineal migration [17], [18], [20], [22]. We found that in *sox2* morphants, the Nodal pathway (as judged by *pitx2* expression) is bilaterally active in approximately 40% of embryos (**Figure S11E–H**). Therefore, the data suggest that the right-sided parapineal organs observed in 10–20% of *sox2* morphants are due mainly to the abnormal bilateral activation of the Nodal signaling.

### Suboptimal Doses of *sox2* Morpholinos Result in the Same, Although of Different Severity, Phenotypes

Sox2 is a dose-dependent regulator during development. In mice, small changes in the levels of SOX2 lead to different phenotypic severities [28]. In order to investigate whether different levels of Sox2 can lead to different phenotypes in terms of the zebrafish epithalamic development, we microinjected suboptimal doses of the two morpholinos. In particular, we injected 0.9 ng or 1.8 ng of each morpholino (in total 1.8 ng or 3.6 ng, respectively) and analyze the number of PhRs and the position of the parapineal organ. Notably, microinjections with 0.9 ng of each morpholino result in embryos indistinguishable from their uninjected siblings. Microinjections of 1.8 ng of each morpholino result in a very mild phenotype (similar to embryos injected with 3.5 ng of *sox2*-MO1 or 2 ng of *sox2*-MO2 shown in **Figure S1**).

As discussed above, knockdown of *sox2* (using our standard morpholino doses: 3.5 ng of *sox2*-MO2) results in an almost two-fold increase in the number of PhRs (16 cells in controls and 29 cells in *sox2* morphants). A similar increase in the number of PhRs is observed in embryos injected with suboptimal concentrations of the two morpholinos, as shown in Table 1. Thus, even small changes in the levels of Sox2, which do not cause any major morphological defects, lead to abnormal increase in the number of PhRs.

**Table 1:** Suboptimal doses of *sox2* morpholinos result in increased number of PhRs and abnormal positioning of the parapineal organ.

	Number of PhRs a	Parapineal position b		
		Left	Right	Bilateral
Control	18 (±1.12, n = 16)	100% (n = 34)	0% (n = 0)	0% (n = 0)
*sox2*MO1+*sox2*MO2 (0.9 ng of each)	29 (±1.25, n = 16)	82% (n = 27)	15% (n = 5)	3% (n = 1)
*sox2*MO1*+sox2*MO2 (1.8 ng of each)	36 (±2.3, n = 13)	64% (n = 29)	4% (n = 2)	31% (n = 14)

a Average number of PhRs (± standard error, number of embryos analyzed).

b Percentage of embryos with left, right or bilateral parapineal projections (number of embryos in each category).

The position of the parapineal organ was assessed by analyzing the GFP expression of *Tg(foxd3:GFP)* embryos. The majority of embryos injected with low dose of the two morpholinos (0.9 ng each) have normal left-sided parapineal organ, but approximately 15% of these embryos exhibit reversal of the parapineal. Only one of these embryos had bilateral parapineal projections (3%). In contrast, injections with 1.8 ng of each morpholino result in increased number of embryos with bilateral parapineal projections (31%), whereas only two embryos (4%) had right-sided parapineal organs. Interestingly, increasing doses of the morpholinos lead to increased percentage of embryos with bilateral parapineal projections in the expense of embryos with right-sided parapineals. These data suggest that small changes in the levels of Sox2 lead to randomization of the parapineal organ. At even lower levels of Sox2, parapineal cells loose their cohesiveness and as a result they are more likely to move individually giving rise to the scattered (bilateral) parapineal phenotype.

## Discussion

The relatively simple zebrafish epithalamus is an excellent model to study brain development. All the main processes that are used iteratively during the brain development, such as neurogenesis, differentiation, cell-fate determination, neuronal migration and break of symmetry, can be studied in the epithalamus. Furthermore, the molecular mechanisms revealed in epithalamic development provide a valuable insight into the complex processes of brain development.

### Sox2 is Essential for Zebrafish Development

We used *sox2* morphant zebrafish as an animal model. This approach has been widely used to recapitulate many aspects of human disease. Lower morpholino doses recapitulate the previously described *sox2* morphant models [39]. At the dose used for our experiments, which is still lower than those used in other studies [40], [41], the overall phenotypes are more severe. However, this is not due to toxicity, as shown by the rescue experiments and the absence of abnormally high levels of apoptosis at 28 hpf. Instead, the severity correlates with dose and efficiency of *sox2* knockdown. This is not surprising, since Sox2 is known to function in a dose dependent manner [28] and thus even slight changes in the levels of its expression are likely to contribute to a phenotypic change.

*sox2* belongs to the *soxB1* family of genes, along with *sox1a*, *sox1b*, *sox3*, *sox19a* and *sox19b*. Previous studies showed that these genes are often functionally redundant [27], [39]. Also, the expression of these genes, especially *sox2*, *sox3*, *sox19a* and *sox19b*, is largely overlapping (www.zfin.org and [42]). It is therefore possible that the knockdown of *sox2* may affect the expression of the other *soxB1* genes and this in turn may contribute to the phenotypic effects observed. Similarly, Sox2, as a transcription factor, is likely to influence the expression of many other genes and thus not all phenotypes may be directly controlled by Sox2.

### Sox2 and Notch Synergistically Control Neurogenesis within the Pineal Gland

We found that Sox2 is expressed in undifferentiated pineal cells and downregulated as the cells start to differentiate and express Isl1. Additionally, knockdown of *sox2* results in increased neurogenesis. The enlarged pineal anlage (as judged by *flh* expression) between 3–8 ss, along with the increased expression of the proneural gene *ascl1a* at 16 hpf, observed in *sox2* morphants may explain the increased neurogenesis.

Next we investigated the relationship between Sox2 and Notch in neurogenesis. We found that simultaneous inhibition of *sox2* and Notch results in synergistic effect on the number of neurons generated. Synergy can arise when disruption of one gene/pathway increases the sensitivity of the phenotype to the second signal [30]. Thus, the small size of *sox2* morphants may increase their sensitivity to drugs such as DAPT that was used to reduce Notch function. However, inhibition of *sox2* and Notch results in additive (not synergistic) effect in cell-type specification, suggesting that *sox2* morphants are not more sensitive to DAPT.

Synergy also arises when two genes/pathways work through partially independent pathways that converge at a common node [30]. Thus, we propose a model where Sox2 controls neurogenesis by modulating the expression of *flh* that in turn controls the downstream genes such as *ascl1a*, whereas Notch controls the expression of *ascl1a* and *neurog1* (but not *flh*) [15].

### Sox2 is the PhR Prepatterning Signal and Controls the PhR Program Independently of the BMP Pathway

In addition to its role in neurogenesis, Sox2 controls PhR, but not PN cell-fate determination. The simultaneous knockdown of *sox2* and Notch results in additive effect on cell-type specification, suggesting that they work through two independent pathways. In contrast, our data suggest that Sox2 controls the PhR program through a BMP-independent mechanism, since: 1) *sox2* morphants have increased PhRs even at 24 hpf, when BMP is relatively normal and 2) the number of extra PhRs is higher than the number of extra BMP-responsive cells in *sox2* morphants when compared to control siblings, 3) knockdown of both *sox2* compensates for the loss of PhR in embryos with compromised BMP pathway.

Notably, Sox2 is expressed throughout the developing epithalamus and in all pineal progenitor cells. How does it therefore inhibit the PhR program only in a subset of them? In humans, SOX2-associated anophthalmia is a haploinsufficiency disease [43]. Also, subtle dosage adjustment was required in the gene-targeted mouse to recapitulate a similar phenotype [28]. Finely tuned function is therefore elicited by precise dosage control and stoichiometry. Alternatively, Sox2 may interact with a partner protein that is expressed only in a subset of progenitor cells (the ones that will become PNs). This is in agreement with previous studies showing that Sox2 requires a partner protein in order to function properly. The partner provides stability and specificity for Sox2 binding to the regulatory region. Thus, distinct partner proteins, expressed in different specific cell types, allow SOX2 to regulate a distinct set of target genes [28].

A number of models can be envisaged to explain the defects observed when Sox2, Notch and/or BMP are compromised. One model involves two modulators controlling the fate of a single cell: one induces the specific fate, whereas the other inhibits the opposite fate. BMP is the PhR-inducing, while Sox2 is the PhR-inhibiting, and Notch the PN-inhibiting modulator. Although this model can explain most of the defects observed in mutants/morphants, it has limitations. For example, simultaneous inhibition of both Notch and BMP does not result in PN-only pineal glands (unpublished data discussed in [44]).

**Figure 9:**
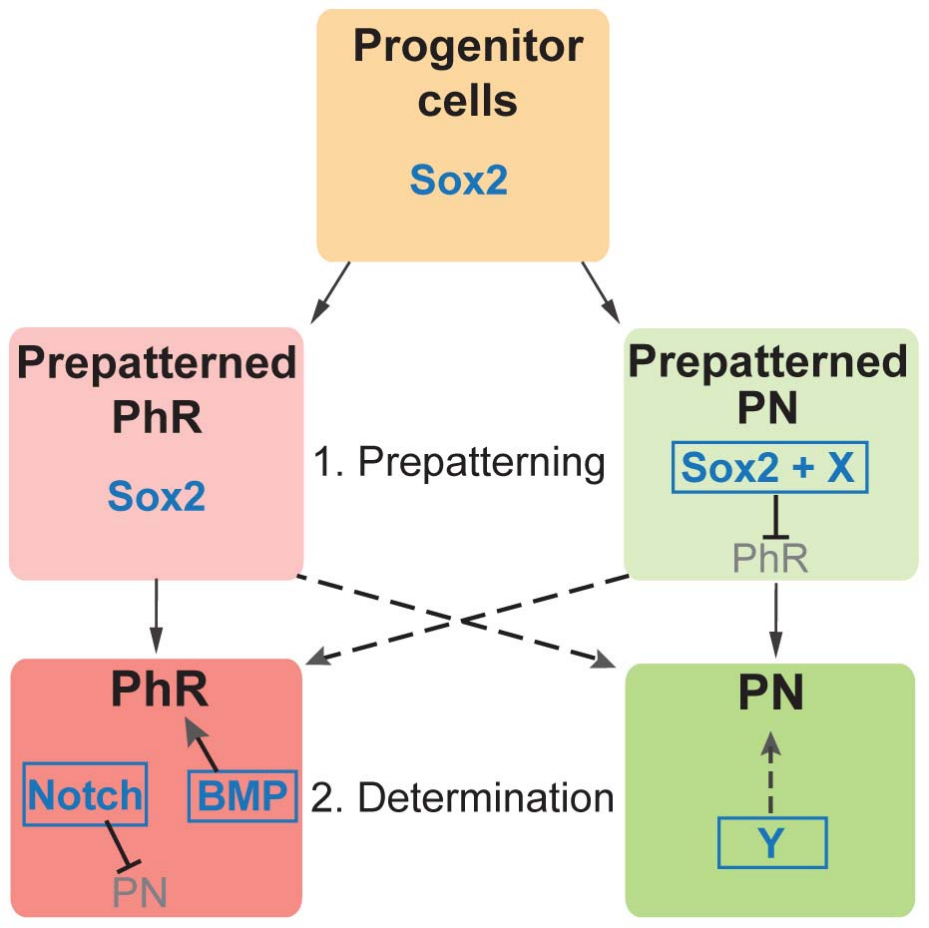
A model for pineal cell-fate determination. Cell-fate determination has two phases: a prepatterning phase, followed by a determination phase. Sox2 (Sox2 levels or Sox2 along with a partner protein (X)) is important during the prepatterning phase, where is inhibits cells from adopting a PhR fate. In contrast, the Notch and BMP pathways are important during the determination fate, where BMP induces the PhR fate and Notch inhibits the PN fate. A yet-to-be-identified modulator (Y) is responsible for inducing the PN fate.

A better model, based on the proposal from Quillien et al. [16], is that pineal progenitor cells are prepatterned (**Figure 9**). A subset of the progenitor cells would be primed to become PhRs, whereas others would be primed to become PNs. The prepatterning would not be sufficient to determine the cell fates. Instead, a second level of regulation (mediated by Notch and BMP) would be necessary to fine-tune it, by mechanisms similar to the ones described on the first model. Our data suggest that Sox2 inhibits the PhR prepatterning in some pineal cells. Therefore, in the absence of Sox2 more or even all cells are prepatterned towards the PhR program. The presence of Notch and BMP ensures that correct number of PhRs and PNs are generated. However, in the extra neurons observed in *sox2* morphants, insufficient Notch is present to inhibit them to become PhRs. As a result, we observed an increased number of PhRs, while the number of PNs is unaffected. Interestingly, a significant increase in the number of BMP-responsive cells is observed in *sox2* morphants, following the increase in the number of *aanat2*-positive cells (PhRs). These data suggest that prepatterning towards the PhR fate may be necessary for the activation of the BMP pathway in the cells that will become PhRs. Therefore, in the case of *sox2* morphants, more cells are prepatterned towards the PhR program and BMP activity is upregulated in order to properly specify these cells.

### Sox2 is Required for Parapineal Development and Habenular Asymmetry

**Figure 10:**
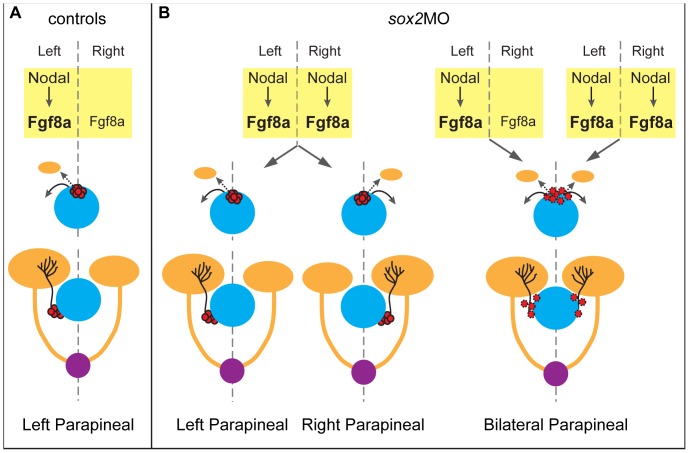
A model for parapineal organ and the habenulae defects in *sox2* morphants. (**A**) In control embryos, the Nodal signaling is activated on the left side of the diencephalon and leads to the upregulation of the bilaterally expressed fgf8a on the left side. The Nodal and fgf8a signals are important for the leftward migration of the parapineal organ, which specifically innervates the left habenula. As a result, the left habenula is larger than the right. (**B**) In a subset of *sox2* morphants, Nodal signaling is bilaterally active leading to randomization of the parapineal laterality and consequently randomization of the habenular asymmetries. In addition, some *sox2* morphants have bilateral parapineal organs, possibly due to abnormal cell-adhesion properties. yellow = diencephalon, blue = pineal gland, red = parapineal cells (dotted lines surrounding parapineal cells represent defective cell-adhesion), orange = habenulae and their projections, purple = interpeduncular nucleus; the main habenular target.

We also found that *sox2* morphants have abnormal parapineal development and disrupted habenular asymmetries. Our data suggest that Sox2 is important for the proper unilateral activation of the Nodal pathway and, as a result, the correct laterality of the parapineal organ and the habenular asymmetries are not achieved in a subset of morphant embryos (**Figure 10**). Notably, the asymmetric activation of Nodal in the lateral plate mesoderm, which is known to be important for the proper laterality of the viscera and for the proper activation of Nodal in the diencephalon, is also defective in *sox2* morphants (data not shown). This suggests that knockdown of *sox2* may also affect the laterality of the visceral organs. Further investigation is required to fully understand the mechanism by which Sox2 controls laterality of the brain and possibly of the body.

About 50% of *sox2* morphants exhibit a rare defect: scattered parapineal cells that are able to project to both the left and right habenulae, resulting in symmetric development of the latter (**Figure 10**). The high frequency of the scattering phenotype makes *sox2* morphants an attractive model to study the mechanisms involved in the migration of parapineal cells as a coherent structure.

Parapineal migration is a collective-cell migration, since a few cells start the migration and are followed by the remaining cells (leader-follower mechanism) [21], while keeping contact points. Collective migration is a very dynamic process that requires proper use of the cytoskeleton, establishment of different cell states (leader/follower cells) and interactions between the migrating cells with each other and with the environment [45]. In the case of parapineal cells, Fgf and Nodal signals reflect interactions between the parapineal cells and the environment. On the other hand, *tbx2b* is important for the specification of leader cells [21]. Interestingly, knockdown of *sox2* leads to a reduction in *tbx2b* expression and this may contribute to the scattering parapineal phenotype. In addition, we hypothesize that Sox2 might be important for the establishment and/or maintenance of communication between the parapineal cells, by modulating cell adhesion. A defective cohesiveness, along with the fact that *fgf8a*, a chemoattractant, is expressed in a broader domain in *sox2* morphants may also lead to the individual migration of parapineal cells towards both the left and the right side of the brain. The cadherin molecule Cdh2 is a good candidate to mediate parapineal cohesiveness, since it is expressed in the epithalamus and controls the adhesion properties of the pineal complex [21]. In agreement with our hypothesis, previous studies demonstrated a role for Sox2 in coordinating the migration of cells by controlling the expression and localization of Cdh2 in rodents and chickens [46], [47].

In conclusion, our findings demonstrate the complexity of neural development and reinforce the excellence of the epithalamus as a model to dissect essential molecular and cellular phenomena. They also show that Sox2 has pleiotropic effects during the development of a single brain structure.

## Materials and Methods

### Animals

All zebrafish procedures were performed in accordance with UK Home office regulation and approved by the University of Edinburgh Ethical committee.

### Strains and Developmental Conditions

An out-crossed wildtype population and the transgenic lines *Tg(elavl3:GFP)*, *Tg(aanat2:GFP)*, *Tg(flh:GFP)*, *Tg(hs:Gal4)*, *Tg(UAS:Notch-intra)*, *Tg(BRE:GFP)* that expresses the destabilized form of GFP, *Tg(foxd3:GFP)* and *Tg(csl:venus)* (unpublished, obtained from Dr. Gering, Nottingham) were used [18], [29], [32], [48]–[52]. Fish were maintained according to [27], [53], [54] and embryos were staged according to Kimmel et al. [55]. Two morpholinos, *sox2*-MO1: CTCGGTTTCCATCATGTTATACATT and *sox2*-MO2: TCTTGAAAGTCTACCCCACCAGCCG, were analyzed (at a final concentration of 3.5 ng, unless otherwise stated) and the phenotype was consistent and fully penetrant. *sox2*-MO2 was used for all the experiments. A standard control morpholino (GeneTools; CCTCTTACCTCAGTTACAATTTATA) was also injected and no phenotype was observed in the overall morphology of the embryos or the number of isl1-positive cells. *Tg(hs:Gal4)*;*Tg(UAS:Notch-intra)* were heat-shocked for 30 minutes at 39°C.

### mRNA Injections

*In vitro* transcribed human *SOX2* mRNA (25 pg) was injected into 1-cell stage wildtype embryos, along with *sox2* morpholino (plasmid obtained from Kleinjan). Microinjections with only *SOX2* mRNA (12.5 pg, 25 pg, 50 pg) were also performed.

### Whole Mount *in situ* Hybridization

Reactions were carried out as previously described [56]. Riboprobes were transcribed from PCR templates amplified from a cDNA library, synthesized from 28 hpf embryos (specific primers available on demand). The *aanat2*, *fgf8a*, *gfi1ab* and *pitx2* probes were transcribed from plasmids (obtained from Klein, Feldman and Blader).

### Western Blot Analysis

Whole embryos were lysed, at 28 hpf, in a modified RIPA buffer (50 mM Tris-Cl, 150 mM NaCl, 1% NP-40, 0.5% sodium deoxycholate, 0.05% SDS and 2 mM EDTA) containing protease inhibitors cocktail (Roche), 1 mM sodium vanadate, 5 mM sodium fluoride and 10 mM iodoacetamide. The lysates were centrifuged for 10 minutes at 16000 g and the postnuclear supernatants were incubated for 5 minutes at 95°C with Laemmli buffer. The proteins were separated on a 10% SDS-polyacrylamide gel and transferred to a nitrocellulose membrane (Hybond). The blots were blocked in milk for 2 hours and then incubated with anti-Sox2 rabbit polyclonal antibody (ab15830, Abcam) overnight. A HRP-coupled anti-rabbit Ig secondary antibody was used for detection by ECL (GE Healthcare).

The densitometric analysis of the western blot was achieved using the Gel Analyzer tool (Fiji). The relative density of Sox2 bands was normalized to the relative density of the corresponding β-tubulin bands (loading control) in control, *sox2*-MO1 and *sox2*-MO2.

### Drug Treatments

To inhibit pigmentation, dechorionated embryos were transferred to E3 medium/0.003% PTU at 24 hpf [57]. To inhibit Notch signaling, embryos were transferred to E3 medium containing 100 µM DAPT (TOCRIS bioscience) and 1% DMSO at 9 hpf. To inhibit BMP signaling, embryos were transferred in E3 medium containing 30, 40 or 50 µM dorsomorphin (Sigma) and 1% DMSO at 6 hpf. For both DAPT and dorsomorphin, control embryos were incubated in E3 medium with or without 1% DMSO.

### Immunofluorescence

Embryos fixed in 4% PFA were incubated overnight in methanol at −20°C. They were then washed in PBS/0.1% Tween 20 and incubated in blocking solution (1% BSA, 0.1% Triton X-100, 0.05% DMSO and 2% sheep serum in PBS) for 1 hour at 4°C. Primary antibodies were incubated overnight at 4°C, followed by washes using PBS/0.1% Triton X-100 and overnight incubation in 1/1000 Alexa Fluor conjucated secondary antibodies. Embryos were washed with PBS/0.1% Triton X. Antibodies: Sox2 (1/200, Abcam, ab97959), Isl1 (1/200, DSHB, 39.4D5) and Myc (1/400, Abcam, ab9132).

The same protocol was used for phalloidin staining with some modifications. All washes were performed using PBS/2% Triton X-100 and staining was performed by incubating the embryos in 1/200 Alexa Fluor 594 Phalloidin (Invitrogen, A12381) for 2 days, at 4°C.

### Imaging

Nikon AZ100 macroscope was used for colour-brightfield imaging of embryos and the Axioskop 2 fluorescence microscope (Carl Zeiss) was used for imaging the eye sections. The A1R confocal imaging system (Nikon) was used for fluorescent imaging of live or fixed embryos. For timelapse experiments, embryos were anaesthetized using Tricaine and mounted in 0.8% low-melting Agarose. Temperature was set at 28.5°C. Timelapse experiments were carried out for 60 hours and images were acquired every 40 minutes.

### Quantifications and Statistics

Fiji was used as a platform to manually count number of Isl1- or Isl1/GFP-positive cells. Volume quantifications of the habenular neuropils were performed using Volocity (PerkinElmer) as previously described [37]. The asymmetry index (AI) was calculated as the difference between the volume of the right and left neuropils divided by their total volume.

To compare multiple groups of cell counts, a Kruskall-Wallis rank sum test was performed using R. In all cases there was a significant difference between groups (p-value <0.001) (data not shown). Post-hoc pairwise comparisons were then performed using Mann-Whitney U test with Bonferroni correction (using R). For habenular neuropil quantifications, the non-parametric Wilcoxon paired signed-rank test was used (http://www.stattools.net/Wilcoxon_Pgm.php).

The chi-square test was used to distinguish between additive and synergistic effects. If p-value was significant, the standardized residual was calculated to determine which groups contribute to the difference. Standardized residual was calculated as the difference between observed and expected frequencies, divided by the square root of the expected frequency. A group contributes to the significant chi-square if the absolute standardized residual value is greater than 2.

## Supporting Information

Figure S1 **The severity of the phenotypes observed in** ***sox2*** **morphants varies between different morpholinos and it is concentration dependent.** (**A**) Lateral view of a control embryo at 3 dpf. (**B**) Microinjections with 3.5 ng *sox2*-MO1 morpholino result in a mild phenotype. (**C**) Microinjections with 3.5 ng *sox2*-MO2 result in a more severe phenotype. (**D**) Microinjections with 2 ng of *sox2*-MO2 result in a mild phenotype. See also Figure 1.
(TIF)

Figure S2 **sox2 modulates neurogenesis within the pineal gland.** (**A–C**) *isl1* is expressed in epiphysial neuronal cells at 28, 32 and 48 hpf. (**D–F**) Knockdown of sox2 results in increased *isl1* expression, suggesting an increase in neurogenesis. See also **Figure 3**.
(TIF)

Figure S3 ***ascl1a*** **is upregulated in** ***sox2*** **morphants.** (**A–F**) Between 16 and 28 hpf, *flh* expression is indistinguishable between control (**A–C**) and *sox2* morphant siblings (**D–F**). (**G–H**) Similarly, no difference was observed in GFP expression of the *Tg(flh:GFP)* (green) between control and *sox2* morphant embryos at 28 hpf. Isl1 (red) was used to mark the pineal gland. (**I–J**) *ascl1a* is expressed within the presumptive pineal gland (red arrows and circles) at 16 hpf in control embryos. (**K–L**) In *sox2* morphants, *ascl1a* expression is upregulated at 16 hpf. Developmental stages are shown at the bottom of each column, scale bars = 25 µm, (**A–H, J, L**) Dorsal views, (**I,K**) Lateral views, (**G,H**) confocal maximum projections. See also **Figure 3**.
(TIF)

Figure S4 **sox2 controls the PhR cell fate.** (**A–D**) *aanat2* is expressed in the photoreceptors between 24 and 48 hpf, as detected by whole mount *in situ* hybridization. (**E–H**) At all stages analyzed, an upregulation of *aanat2* expression is observed in *sox2* morphants when compared to control siblings. (**I–K**) *pax6b* is expressed in the projection neurons and a subset of pineal precursors from 28 hpf to 48 hpf. (**L–N**) Downregulation of sox2 does not affect the number of *pax6b*-positive cells. At 48 hpf, *pax6b* is expressed in a broader domain in *sox2* morphants when compared to control siblings. Developmental stages are shown at the bottom of each column, scale bars = 25 µm. See also **Figure 4**.
(TIF)

Figure S5 **Downregulation of sox2 does not affect Notch activity within the pineal gland.** (**A–C**) *Tg(csl:venus)* is a Notch reporter line and drives venus expression (**B**) within the pineal gland, which is marked by isl1 antibody staining (**A**). Only about 50% of venus-expressing cells are positive for isl1. (**D–F**) Microinjections with *sox2* morpholinos have no effect on Notch activity, as shown by venus expression. (**G**) Average number of total venus-positive cells (venus^+^/isl1^+^ and venus^+^/isl^−^) in control (purple bars) and *sox2* morphants (orange bars) at 24 and 28 hpf. Confocal maximum projections, scale bars = 25 µm, error bars represent ± standard error, MWU test; not significant; p-value >0.05, number of embryos counted is shown in each bar. See also **Figure 5**, **Figure S6–S8** and **Movie S1–S2**.
(TIF)

Figure S6 **Downregulation of Notch results in more isl1^+^/sox2** ^−^ **cells in relation to controls.** (**A–E’’**) sox2 is expressed throughout the pineal anlage and is downregulated in isl1-positive cells, in DMSO-treated control embryos at 15 ss. (**A–E**) sox2 expression, (**A’–E’**) isl1 expression, (**A’’–E’’**) merged images of sox2, isl1 and *Tg(flh:GFP)* that marks the pineal anlage. (**F’–J’’**) DAPT treatment results in increased number of cells expressing isl1 (**F’–J’**). sox2 (**F–J**) is still expressed in the undifferentiated pineal precursor cells (green in **F’’–J’’**) but not in the differentiated isl1-positive cells (**F’–J’**). Since there are more isl1-positive cells, sox2 is downregulated in a broader domain in relation to controls. Series of optical sections from dorsal (first column) to ventral (5^th^ column) obtained using confocal microscope, scale bars = 25 µm, yellow arrows show cells in which sox2 is downregulated and isl1 is upregulated, insets show a three times magnified view of the image. See also **Figure 5**, **Movie S1**.
(TIF)

Figure S7 **Upregulation of Notch results in a broader domain of sox2 expression, at 20 ss.** (**A–D’’**) sox2 is expressed throughout the pineal anlage and is downregulated in isl1-positive cells, in heat-shocked control embryos, at 20 ss. (**A–D**) sox2 expression, (**A’–D’**) isl1 expression, (**A’’–D’’**) merged images of sox2, isl1 and myc-tag, showing that heat-shock did not activate the *Notch1a* intracellular domain. (**E–H’’**) Heat-shock of the double transgenics *Tg(hs:Gal4)*; *Tg(UAS:Notch-intra)* results in fewer isl1-positive cells (**E’–H’**) and therefore sox2 is expressed in a broader domain (**E–H**). (**E’’–H’’**) Merged images of sox2, isl1 and myc-tag, showing the activation of the transgene and thus the upregulation of Notch. Series of optical sections from dorsal (first column) to ventral (4^th^ column) obtained using confocal microscope, scale bars = 25 µm, yellow arrows show cells in which sox2 is downregulated and isl1 is upregulated, insets show a three times magnified view of the image. See also **Figure 5** **, Movie S2**.
(TIF)

Figure S8 **Downregulation of BMP does not affect sox2 expression at 15 ss.** (**A–E’’**) sox2 is expressed throughout the pineal anlage and is downregulated in isl1-positive cells, in DMSO-treated control embryos. (**A–E**) sox2 expression, (**A’–E’**) isl1 expression, (**A’’–E’’**) merged images of sox2, isl1 and *Tg(flh:GFP)*, showing the presumptive pineal gland. (**F–J’’**) Dorsomorphin treatment (40 µM) does not affect sox2 expression at 15 ss. sox2 is expressed in the pineal precursors and is downregulated with differentiation (as shown by isl1-positive cells). (**F–J**) sox2 expression, (**F’–J’**) isl1 expression, (**F’’–J’’**) merged images of sox2, isl1 and *Tg(flh:GFP)*. Series of optical sections from dorsal (first column) to ventral (5^th^ column) obtained using confocal microscope, scale bars = 25 µm, yellow arrows show cells in which sox2 is downregulated and isl1 is upregulated, insets show a three times magnified view of the image. See also **Figure 6** **, Movie S3**.
(TIF)

Figure S9 **Simultaneous knockdown of** ***sox2*** **and BMP rescues the number of PhRs.** (**A**) DMSO-treated control embryos at 28 hpf, with PhR in green (*Tg(aanat2:GFP)*) and Sox2 in red. (**B**) Dorsomorphin treatment, that inhibits the BMP pathway, leads to a reduced number of PhRs, while Sox2 expression is normal. (**C**) Knockdown of *sox2* leads to increased number of PhRs. (**D**) Simultaneous inhibition of *sox2* and BMP results in a number of PhR similar to the DMSO-treated control siblings. (**E**) Average number of PhR in DMSO-treated (purple bar), dorsomorphin-treated (green bar), *sox2* morphant treated with DMSO (orange bar) and *sox2* morphant treated with dorsomorphin (red bar) embryos. Confocal maximum projections of 28 hpf embryos, error bars represent ± standard error, * = p-value <0.05 (MWU test), ** = p-value <0.001 (MWU test).
(TIF)

Figure S10 ***tbx2b*** **is downregulated in** ***sox2*** **morphants.** (**A–C**) *tbx2b* is expressed within the pineal gland anlage and is important for the proper specification of parapineal cells. (**D–F**) Downregulation of sox2 results in reduced *tbx2b* expression at all stages analyzed. Developmental stages are shown at the bottom of each column, scale bars = 25 µm. See also **Figure 7** **–** **8** and **Movie S4**.
(TIF)

Figure S11 ***fgf8a*** **and Nodal activity are disrupted in** ***sox2*** **morphants.** (**A–D’**) *fgf8a* is normally expressed bilaterally (although higher expression is detected on the left than the right) in the epithalamus, in control embryos (**A–A’**). As development proceeds, *fgf8a* expression becomes restricted to the medial part of the diencephalon (**C–C’**). At early stages, *fgf8a* expression is normal, in *sox2* morphants (**B–B’**). However, at 3 dpf, *fgf8a*-positive cells are found in a broader domain when compared to control siblings (red brackets and arrows) (**C–D’**). (**E–E’**) *pitx2* is normally expressed in the left side of zebrafish diencephalon (red arrow). (**F–F’**) Approximately 60% of *sox2* morphants have normal left-sided *pitx2* expression, whereas (**G–G’**) 40% of embryos have abnormal bilateral *pitx2* expression (yellow arrows show abnormal right-sided expression). (**H**) Average percentage of embryos with left or bilateral *pitx2* expression in controls (purple bars) and *sox2* morphants (orange bars). (**A–G**) Dorsal views, (**A’–G’**) frontal views of the same embryos. Developmental stages are shown at the bottom of each column, scale bars = 25 µm, error bars represent ± standard error. See also **Figure 7** **–** **8** and **Movie S4**.
(TIF)

Movie S1 **Downregulation of Notch results in more isl1^+^/sox2** ^−^ **cells in relation to controls at 15 ss.** sox2 (red) is expressed in undifferentiated GFP-positive (green) pineal cells of *Tg(flh:GFP)* embryos, but it is downregulated in post-mitotic isl1-expressing cells (blue), in DMSO-treated embryos (left panel). DAPT treatment (right panel) leads to increased number of isl-positive cells and thus broader sox2-negative domain. Navigation through the optical stacks starting with the dorsal-most optical section of the pineal anlage and moving ventrally.
(MOV)

Movie S2 **Upregulation of Notch results in a broader domain of sox2 expression at 20 ss.** sox2 (red) is expressed in undifferentiated GFP-positive (green) pineal cells of *Tg(flh:GFP)* embryos, but it is downregulated in post-mitotic isl1-expressing cells (blue), in heat-shocked control embryos (left panel). Heat-shock of *Tg(hs:Gal4);Tg(UAS:Notch-intra)* (right panel) results in upregulation of Notch signaling. Upregulation of Notch leads to fewer isl1 cells and thus sox2 is expressed in a broader domain. Navigation through the optical stacks starting with the dorsal-most optical section of the pineal anlage and moving ventrally.
(MOV)

Movie S3 **Downregulation of BMP does not affect sox2 expression at 15 ss.** sox2 (red) is expressed in undifferentiated GFP-positive (green) pineal cells of *Tg(flh:GFP)* embryos, but it is downregulated in post-mitotic isl1-expressing cells (blue), in DMSO-treated embryos (left panel). Dorsomorphin treatment (right panel), resulting in downregulation of BMP signaling, does not affect sox2 expression. Navigation through the optical stacks, starting with the dorsal-most optical section of the pineal anlage and moving ventrally.
(MOV)

Movie S4 **Downregulation of sox2 results in abnormal parapineal migration.** Timelapse of *Tg(foxd3:GFP)* embryos from 30 hpf until 90 hpf (images were acquired every 40 minutes), showing the migration of parapineal cells towards the left, in control embryos (top left panel). Downregulation of sox2 leads to: normal-left sided migration of parapineal cells (top right panel), migration towards the right (bottom left panel) and scattering of parapineal cells around the pineal gland (bottom right panel).
(MOV)
