# Supplementary figures and images for: Pleiotropic Effects of Sox2 during the Development of the Zebrafish Epithalamus
Source: PLoS One. 2014 Jan 31;9(1):e87546. doi: 10.1371/journal.pone.0087546 (PMC3909122; doi:10.1371/journal.pone.0087546)

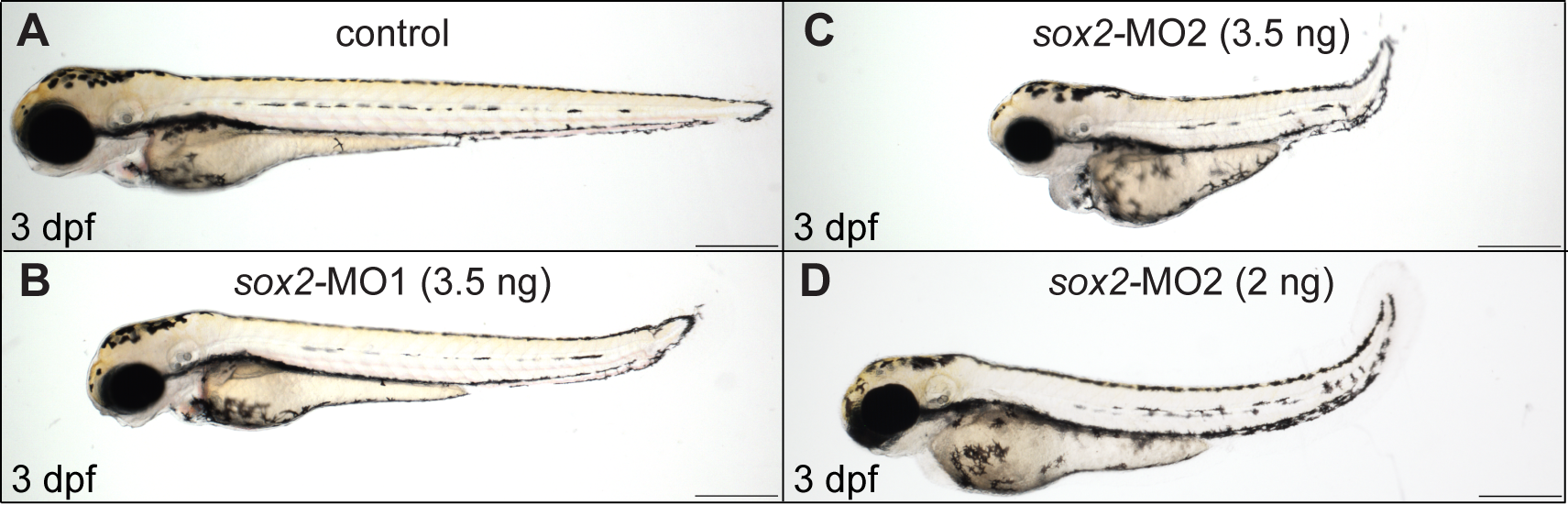

Supplement: Figure S1 — The severity of the phenotypes observed in sox2 morphants varies between different morpholinos and it is concentration dependent. (A) Lateral view of a control embryo at 3 dpf. (B) Microinjections with 3.5 ng sox2-MO1 morpholino result in a mild phenotype. (C) Microinjections with 3.5 ng sox2-MO2 result in a more severe phenotype. (D) Microinjections with 2 ng of sox2-MO2 result in a mild phenotype. See also Figure 1. (TIF) [file pone.0087546.s001.tif]

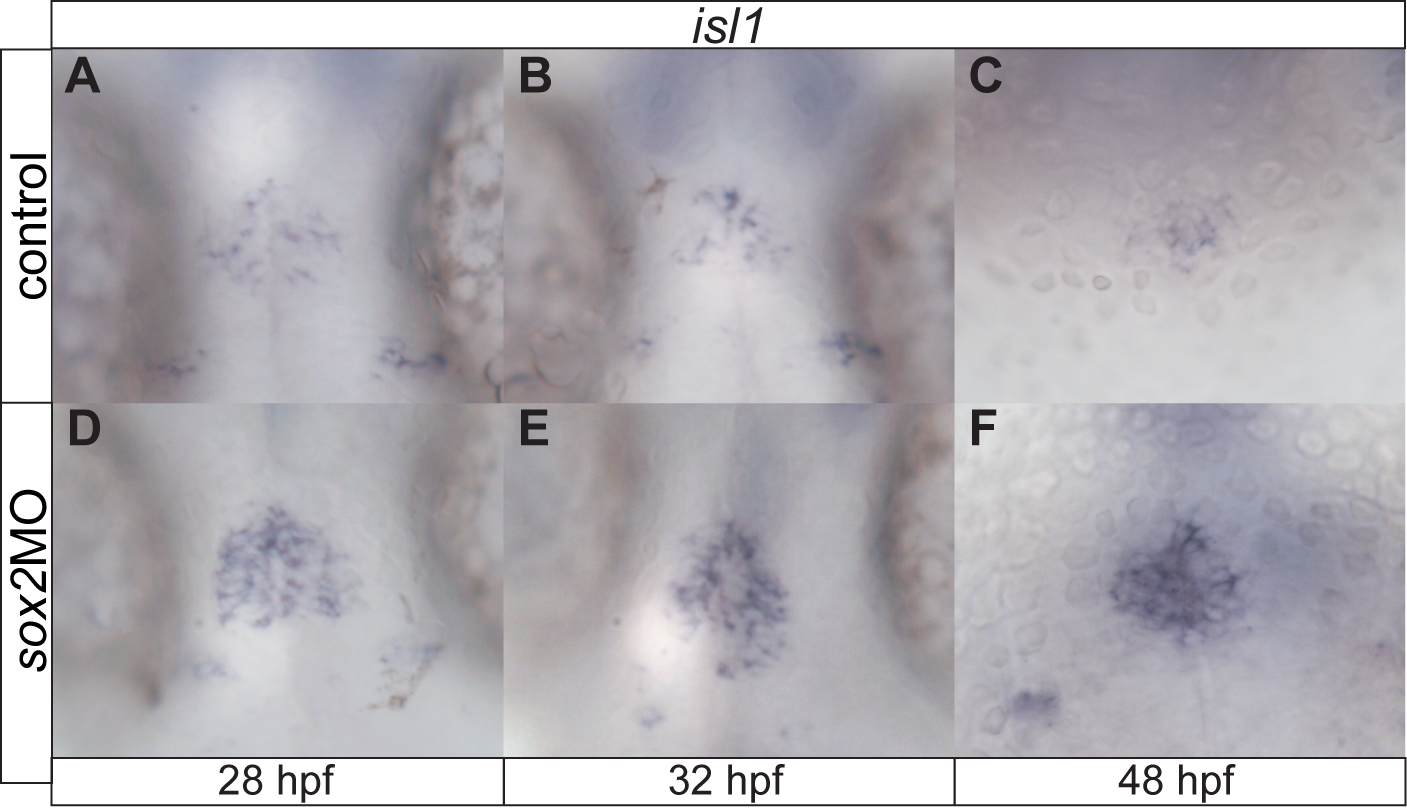

Supplement: Figure S2 — sox2 modulates neurogenesis within the pineal gland. (A–C) isl1 is expressed in epiphysial neuronal cells at 28, 32 and 48 hpf. (D–F) Knockdown of sox2 results in increased isl1 expression, suggesting an increase in neurogenesis. See also Figure 3 . (TIF) [file pone.0087546.s002.tif]

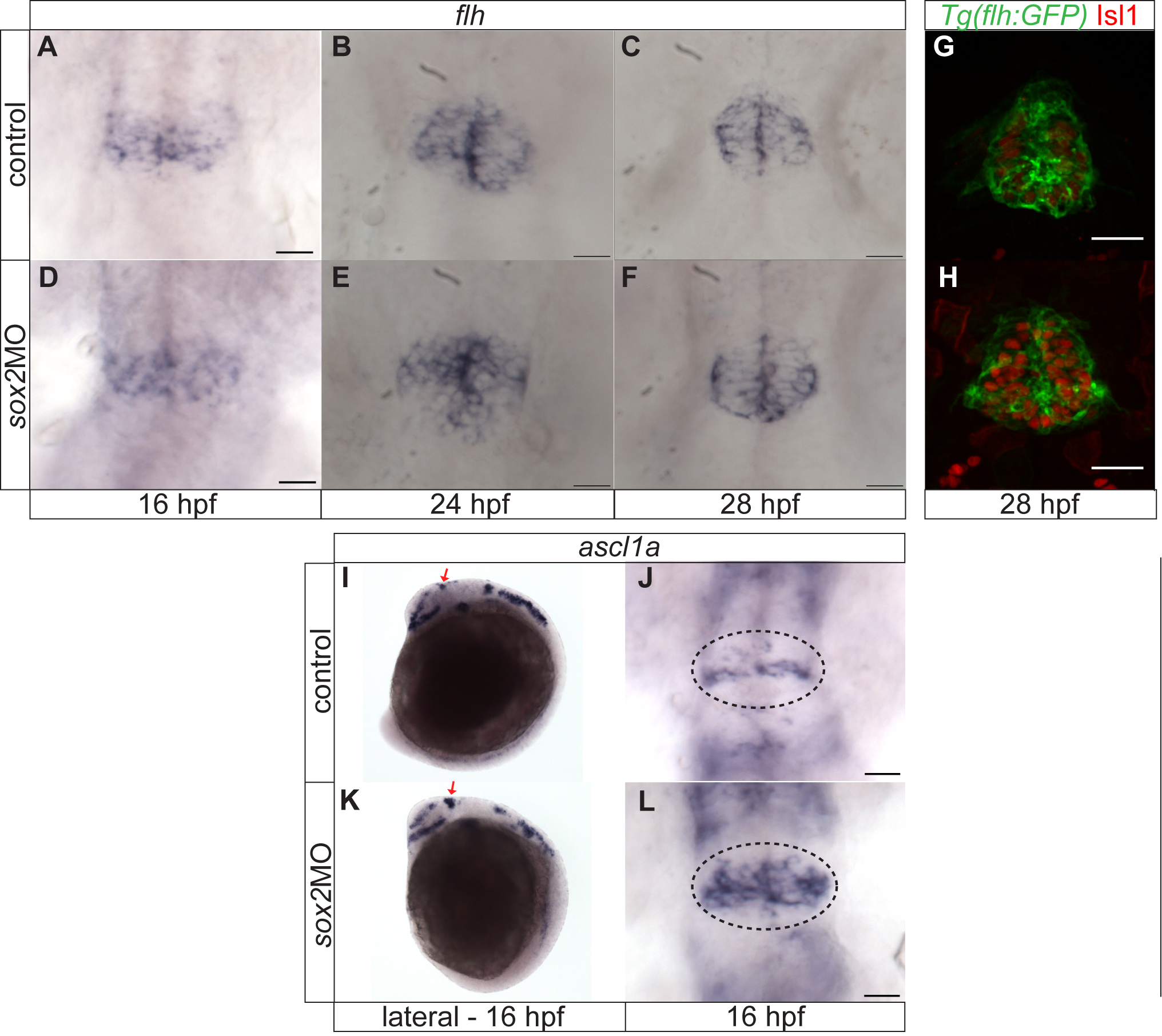

Supplement: Figure S3 — ascl1a is upregulated in sox2 morphants. (A–F) Between 16 and 28 hpf, flh expression is indistinguishable between control (A–C) and sox2 morphant siblings (D–F). (G–H) Similarly, no difference was observed in GFP expression of the Tg(flh:GFP) (green) between control and sox2 morphant embryos at 28 hpf. Isl1 (red) was used to mark the pineal gland. (I–J) ascl1a is expressed within the presumptive pineal gland (red arrows and circles) at 16 hpf in control embryos. (K–L) In sox2 morphants, ascl1a expression is upregulated at 16 hpf. Developmental stages are shown at the bottom of each column, scale bars = 25 µm, (A–H, J, L) Dorsal views, (I,K) Lateral views, (G,H) confocal maximum projections. See also Figure 3 . (TIF) [file pone.0087546.s003.tif]

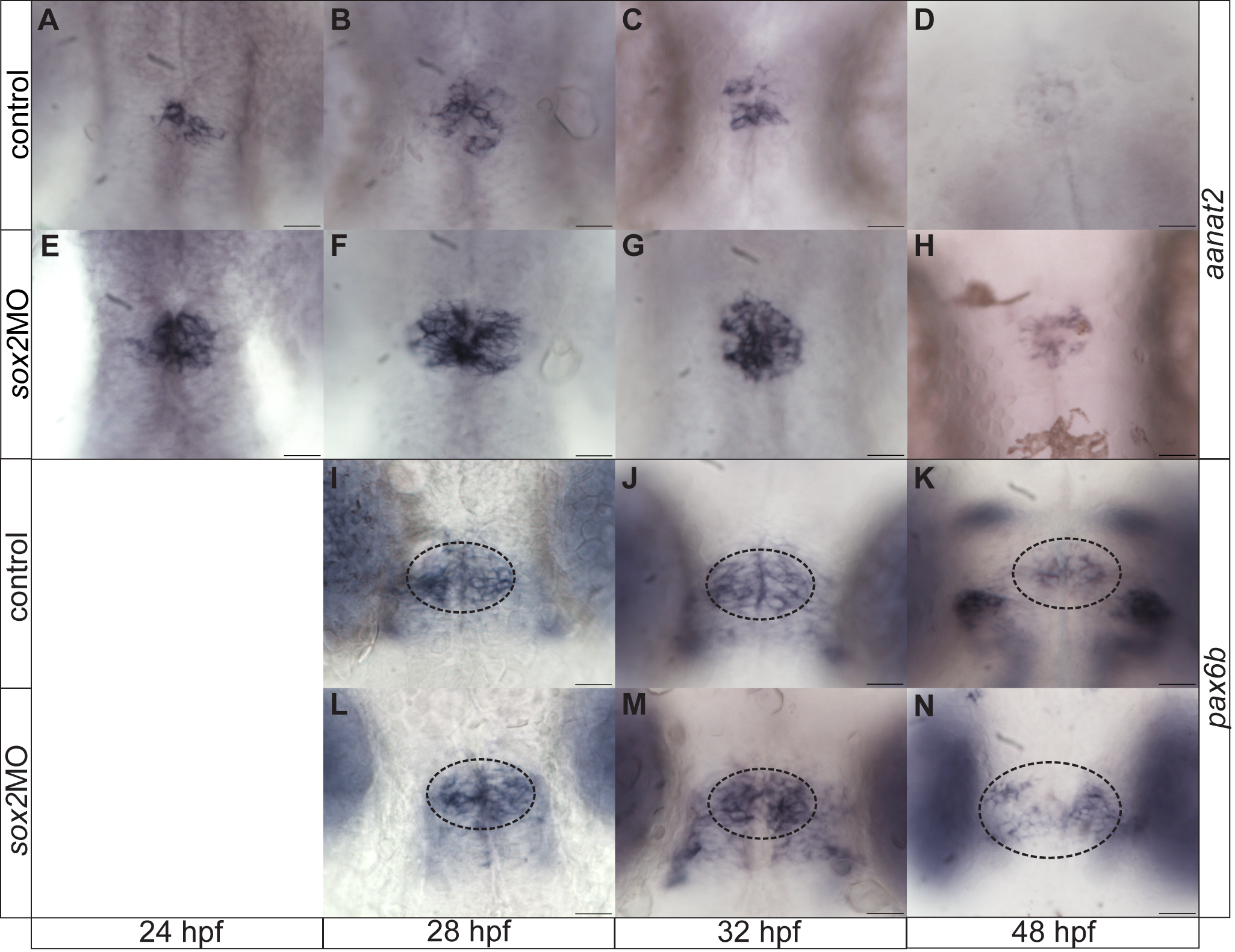

Supplement: Figure S4 — sox2 controls the PhR cell fate. (A–D) aanat2 is expressed in the photoreceptors between 24 and 48 hpf, as detected by whole mount in situ hybridization. (E–H) At all stages analyzed, an upregulation of aanat2 expression is observed in sox2 morphants when compared to control siblings. (I–K) pax6b is expressed in the projection neurons and a subset of pineal precursors from 28 hpf to 48 hpf. (L–N) Downregulation of sox2 does not affect the number of pax6b-positive cells. At 48 hpf, pax6b is expressed in a broader domain in sox2 morphants when compared to control siblings. Developmental stages are shown at the bottom of each column, scale bars = 25 µm. See also Figure 4 . (TIF) [file pone.0087546.s004.tif]

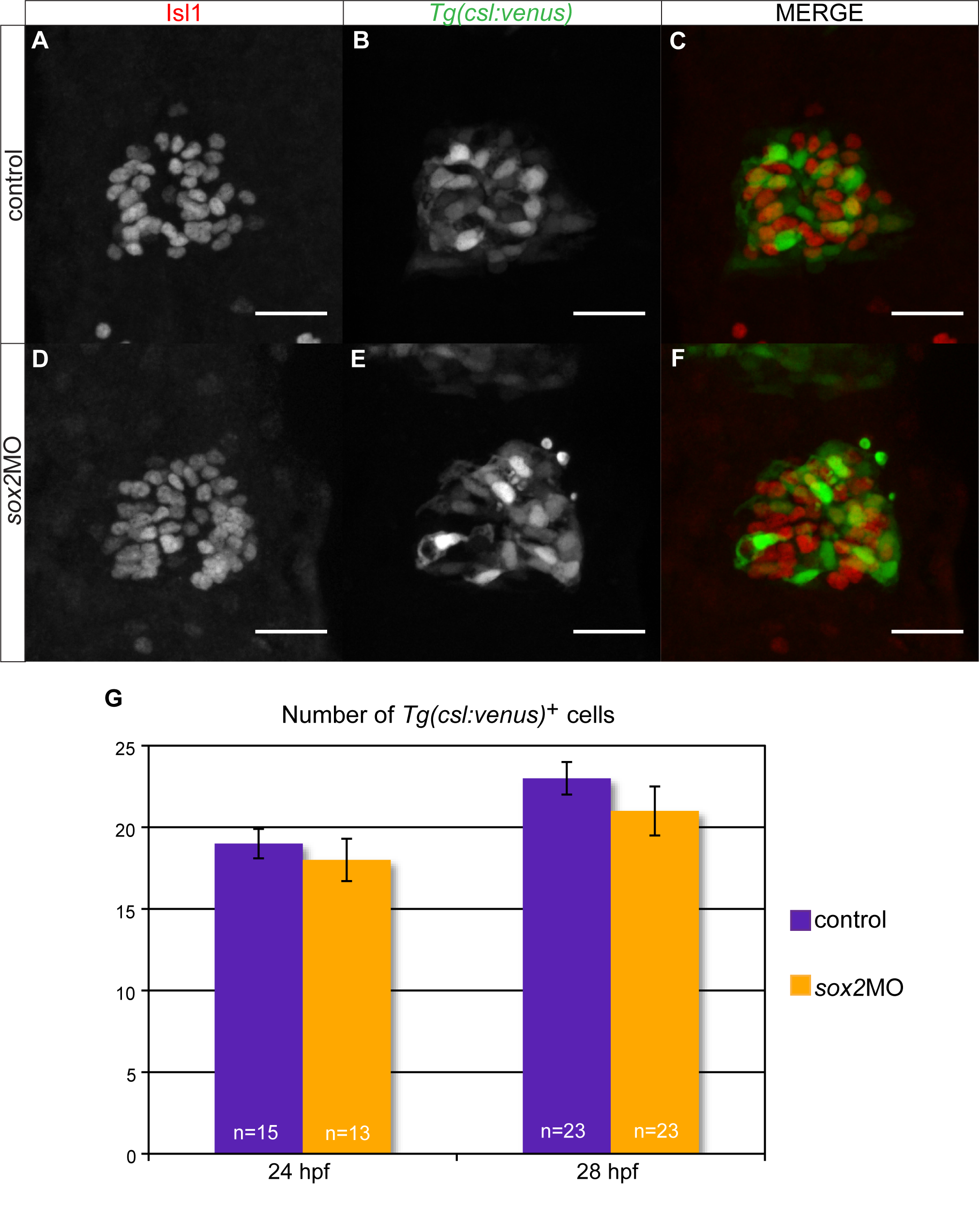

Supplement: Figure S5 — Downregulation of sox2 does not affect Notch activity within the pineal gland. (A–C) Tg(csl:venus) is a Notch reporter line and drives venus expression (B) within the pineal gland, which is marked by isl1 antibody staining (A). Only about 50% of venus-expressing cells are positive for isl1. (D–F) Microinjections with sox2 morpholinos have no effect on Notch activity, as shown by venus expression. (G) Average number of total venus-positive cells (venus+/isl1+ and venus+/isl−) in control (purple bars) and sox2 morphants (orange bars) at 24 and 28 hpf. Confocal maximum projections, scale bars = 25 µm, error bars represent ± standard error, MWU test; not significant; p-value >0.05, number of embryos counted is shown in each bar. See also Figure 5 , Figure S6–S8 and Movie S1–S2. (TIF) [file pone.0087546.s005.tif]

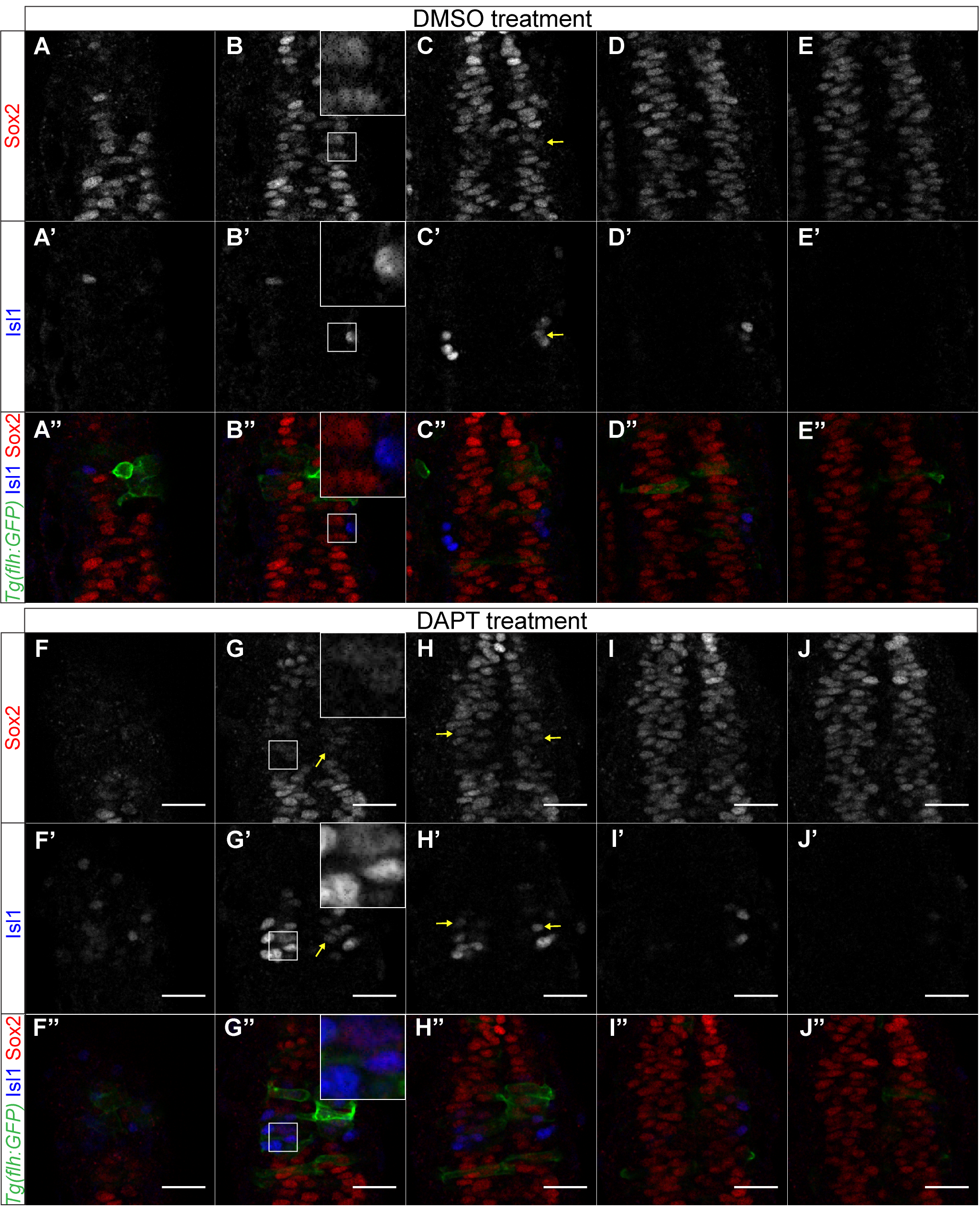

Supplement: Figure S6 — Downregulation of Notch results in more isl1+/sox2 − cells in relation to controls. (A–E’’) sox2 is expressed throughout the pineal anlage and is downregulated in isl1-positive cells, in DMSO-treated control embryos at 15 ss. (A–E) sox2 expression, (A’–E’) isl1 expression, (A’’–E’’) merged images of sox2, isl1 and Tg(flh:GFP) that marks the pineal anlage. (F’–J’’) DAPT treatment results in increased number of cells expressing isl1 (F’–J’). sox2 (F–J) is still expressed in the undifferentiated pineal precursor cells (green in F’’–J’’) but not in the differentiated isl1-positive cells (F’–J’). Since there are more isl1-positive cells, sox2 is downregulated in a broader domain in relation to controls. Series of optical sections from dorsal (first column) to ventral (5th column) obtained using confocal microscope, scale bars = 25 µm, yellow arrows show cells in which sox2 is downregulated and isl1 is upregulated, insets show a three times magnified view of the image. See also Figure 5 , Movie S1. (TIF) [file pone.0087546.s006.tif]

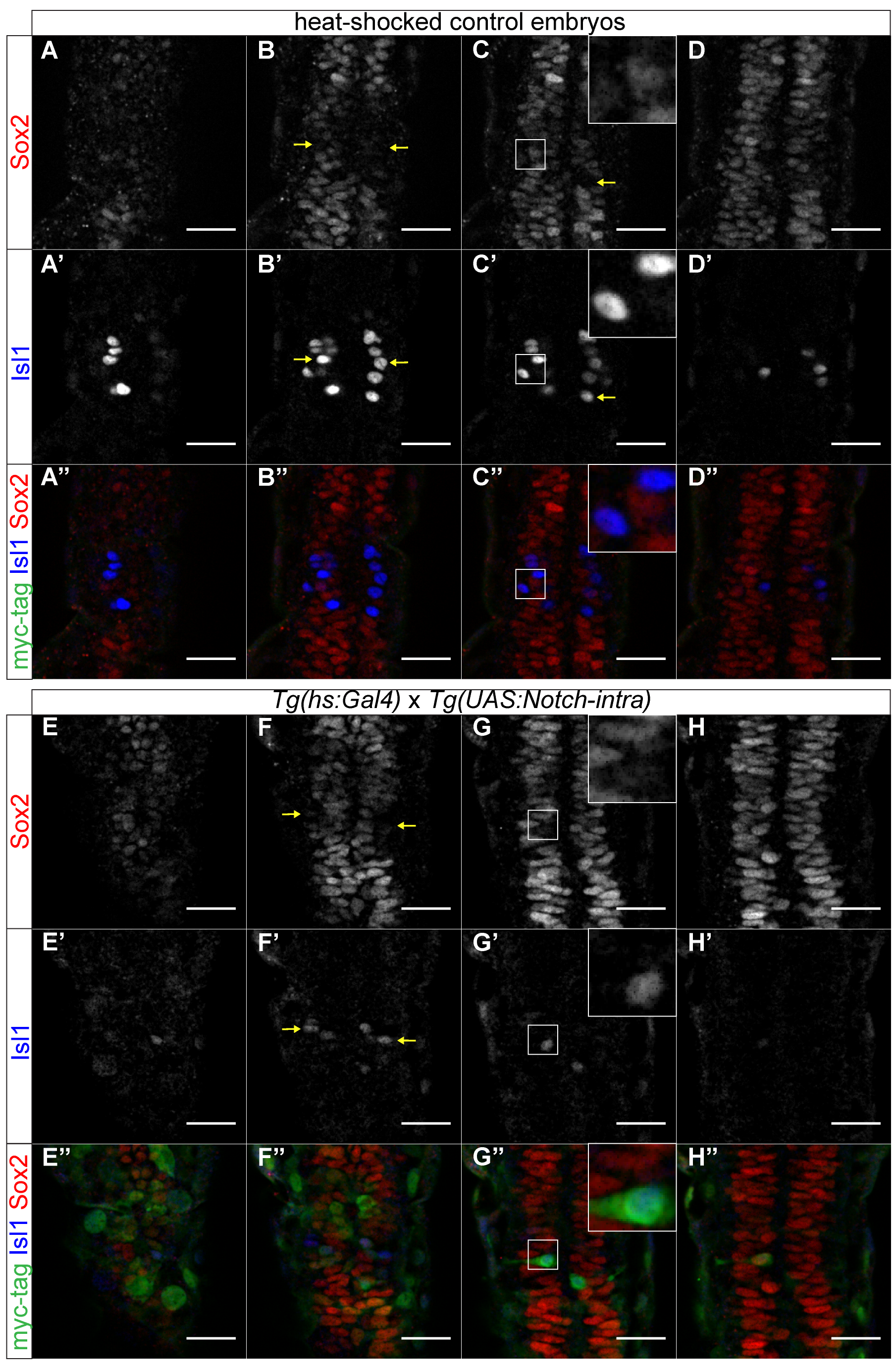

Supplement: Figure S7 — Upregulation of Notch results in a broader domain of sox2 expression, at 20 ss. (A–D’’) sox2 is expressed throughout the pineal anlage and is downregulated in isl1-positive cells, in heat-shocked control embryos, at 20 ss. (A–D) sox2 expression, (A’–D’) isl1 expression, (A’’–D’’) merged images of sox2, isl1 and myc-tag, showing that heat-shock did not activate the Notch1a intracellular domain. (E–H’’) Heat-shock of the double transgenics Tg(hs:Gal4); Tg(UAS:Notch-intra) results in fewer isl1-positive cells (E’–H’) and therefore sox2 is expressed in a broader domain (E–H). (E’’–H’’) Merged images of sox2, isl1 and myc-tag, showing the activation of the transgene and thus the upregulation of Notch. Series of optical sections from dorsal (first column) to ventral (4th column) obtained using confocal microscope, scale bars = 25 µm, yellow arrows show cells in which sox2 is downregulated and isl1 is upregulated, insets show a three times magnified view of the image. See also Figure 5 , Movie S2. (TIF) [file pone.0087546.s007.tif]

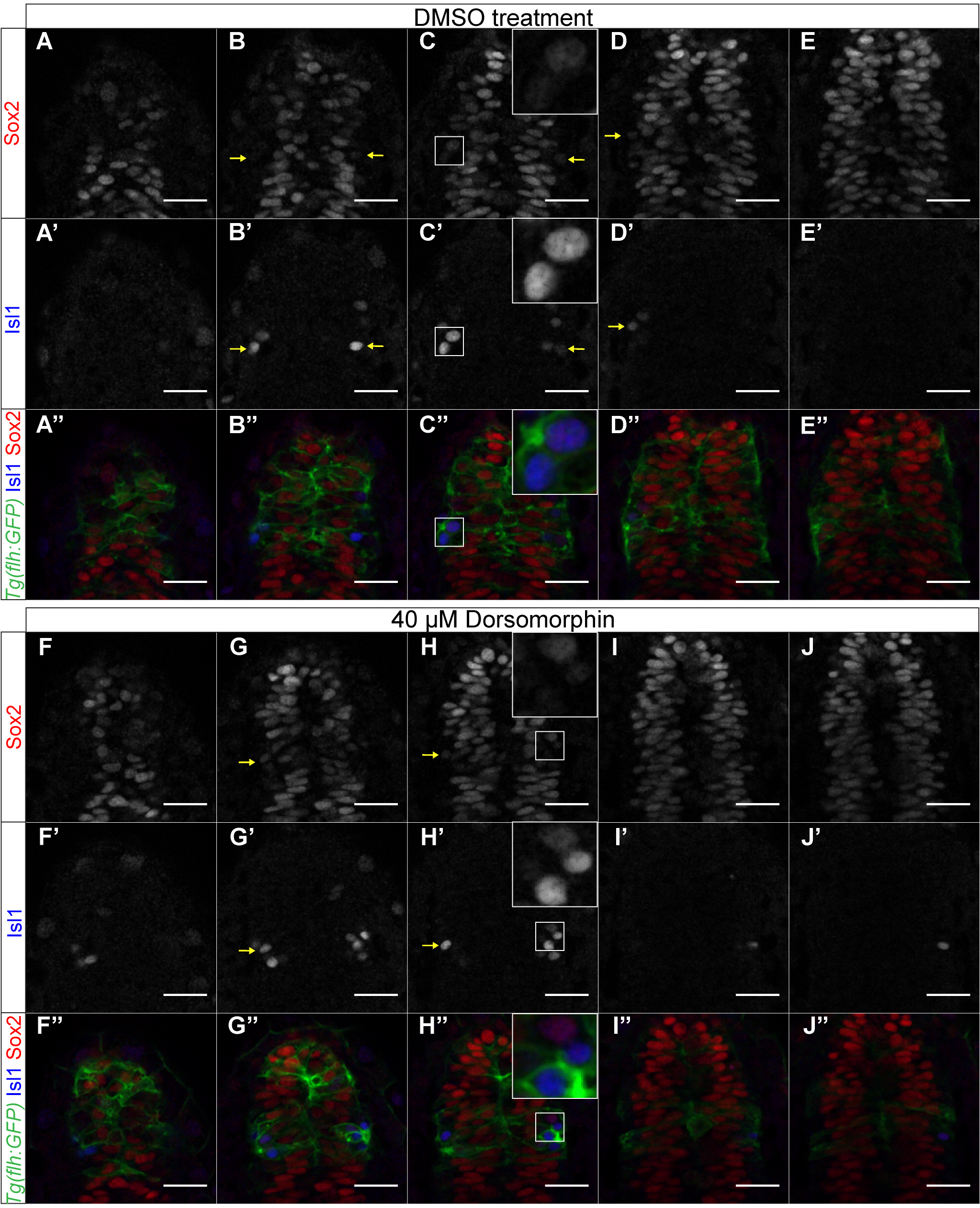

Supplement: Figure S8 — Downregulation of BMP does not affect sox2 expression at 15 ss. (A–E’’) sox2 is expressed throughout the pineal anlage and is downregulated in isl1-positive cells, in DMSO-treated control embryos. (A–E) sox2 expression, (A’–E’) isl1 expression, (A’’–E’’) merged images of sox2, isl1 and Tg(flh:GFP), showing the presumptive pineal gland. (F–J’’) Dorsomorphin treatment (40 µM) does not affect sox2 expression at 15 ss. sox2 is expressed in the pineal precursors and is downregulated with differentiation (as shown by isl1-positive cells). (F–J) sox2 expression, (F’–J’) isl1 expression, (F’’–J’’) merged images of sox2, isl1 and Tg(flh:GFP). Series of optical sections from dorsal (first column) to ventral (5th column) obtained using confocal microscope, scale bars = 25 µm, yellow arrows show cells in which sox2 is downregulated and isl1 is upregulated, insets show a three times magnified view of the image. See also Figure 6 , Movie S3. (TIF) [file pone.0087546.s008.tif]

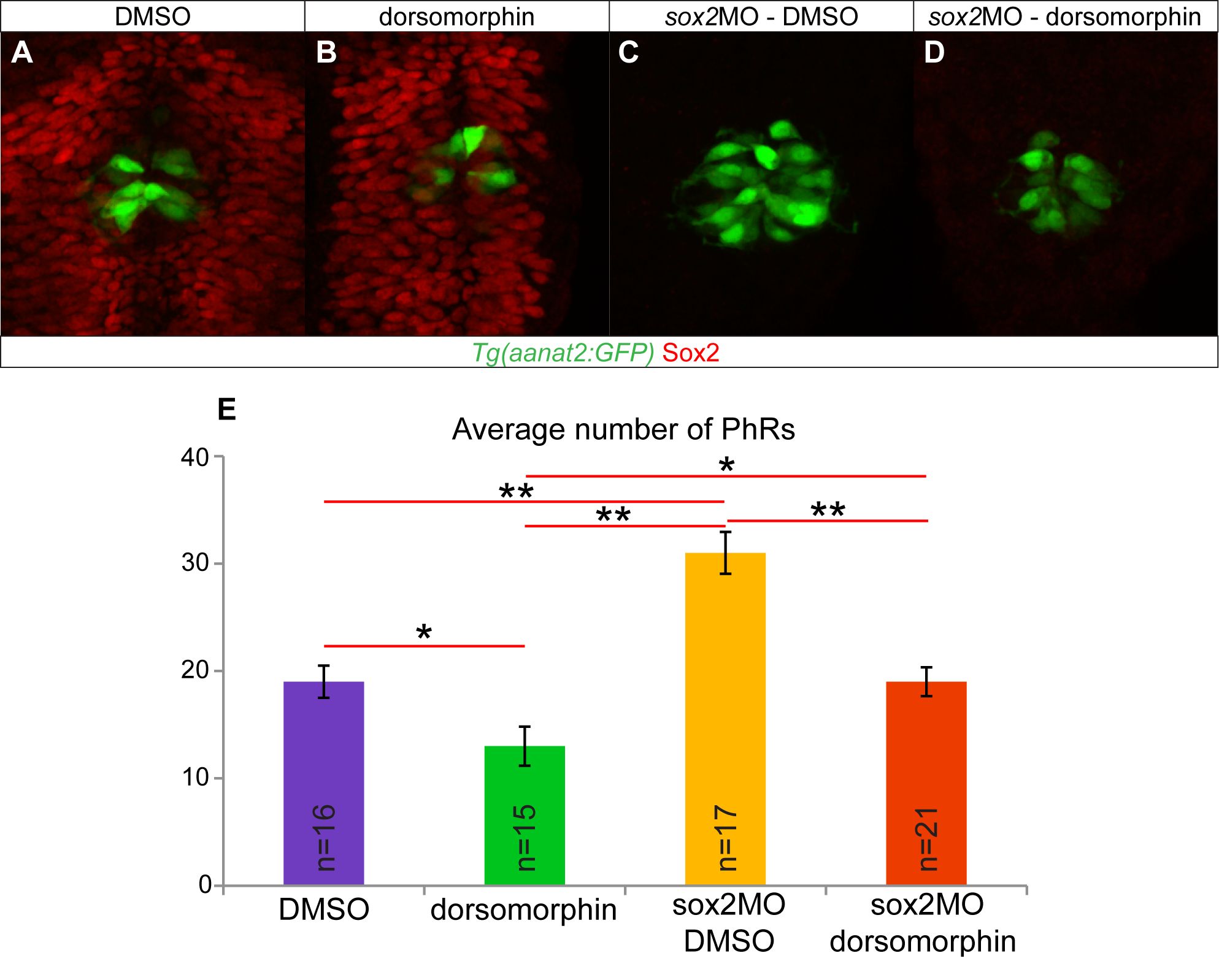

Supplement: Figure S9 — Simultaneous knockdown of sox2 and BMP rescues the number of PhRs. (A) DMSO-treated control embryos at 28 hpf, with PhR in green (Tg(aanat2:GFP)) and Sox2 in red. (B) Dorsomorphin treatment, that inhibits the BMP pathway, leads to a reduced number of PhRs, while Sox2 expression is normal. (C) Knockdown of sox2 leads to increased number of PhRs. (D) Simultaneous inhibition of sox2 and BMP results in a number of PhR similar to the DMSO-treated control siblings. (E) Average number of PhR in DMSO-treated (purple bar), dorsomorphin-treated (green bar), sox2 morphant treated with DMSO (orange bar) and sox2 morphant treated with dorsomorphin (red bar) embryos. Confocal maximum projections of 28 hpf embryos, error bars represent ± standard error, * = p-value <0.05 (MWU test), ** = p-value <0.001 (MWU test). (TIF) [file pone.0087546.s009.tif]

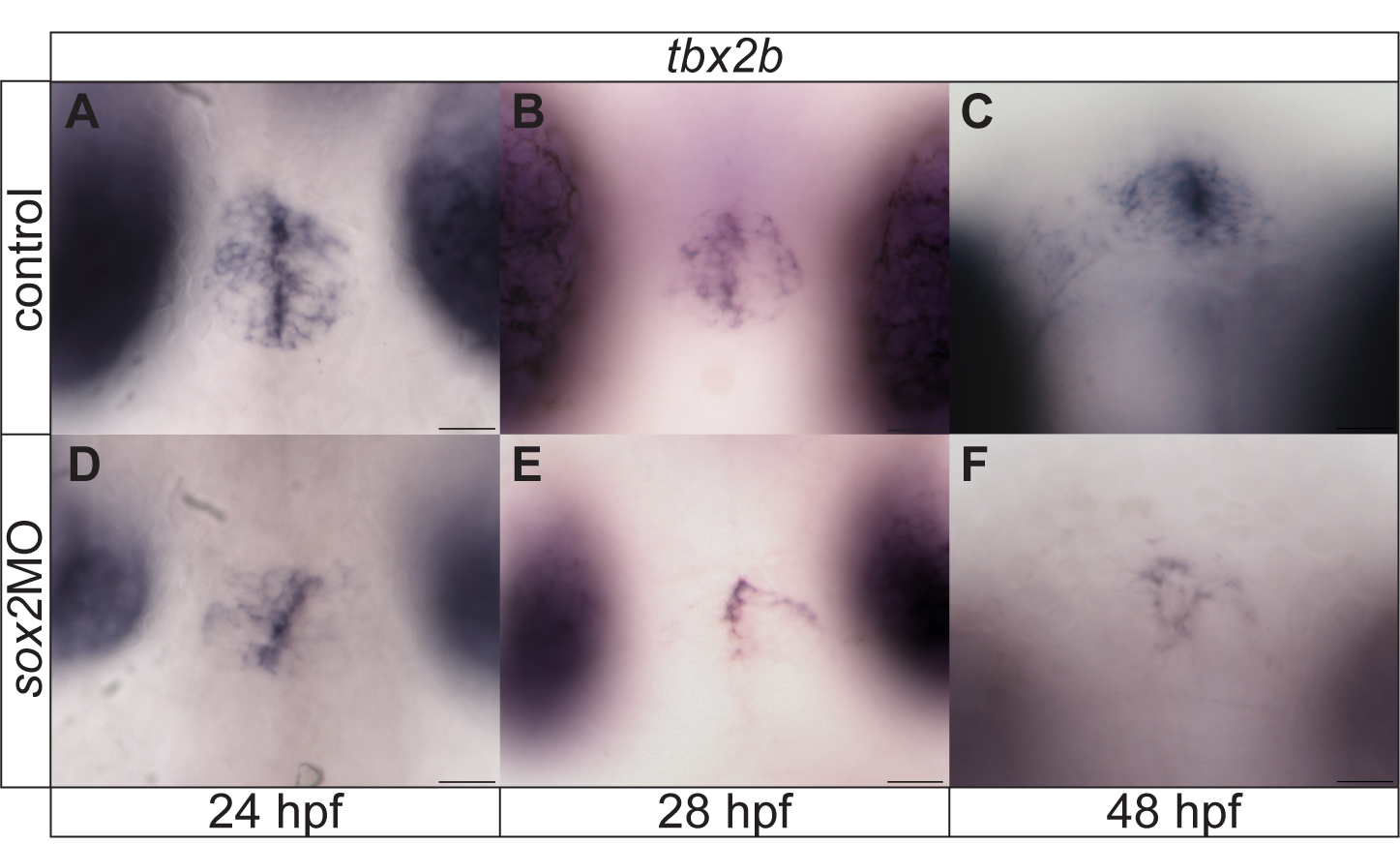

Supplement: Figure S10 — tbx2b is downregulated in sox2 morphants. (A–C) tbx2b is expressed within the pineal gland anlage and is important for the proper specification of parapineal cells. (D–F) Downregulation of sox2 results in reduced tbx2b expression at all stages analyzed. Developmental stages are shown at the bottom of each column, scale bars = 25 µm. See also Figure 7 – 8 and Movie S4. (TIF) [file pone.0087546.s010.tif]

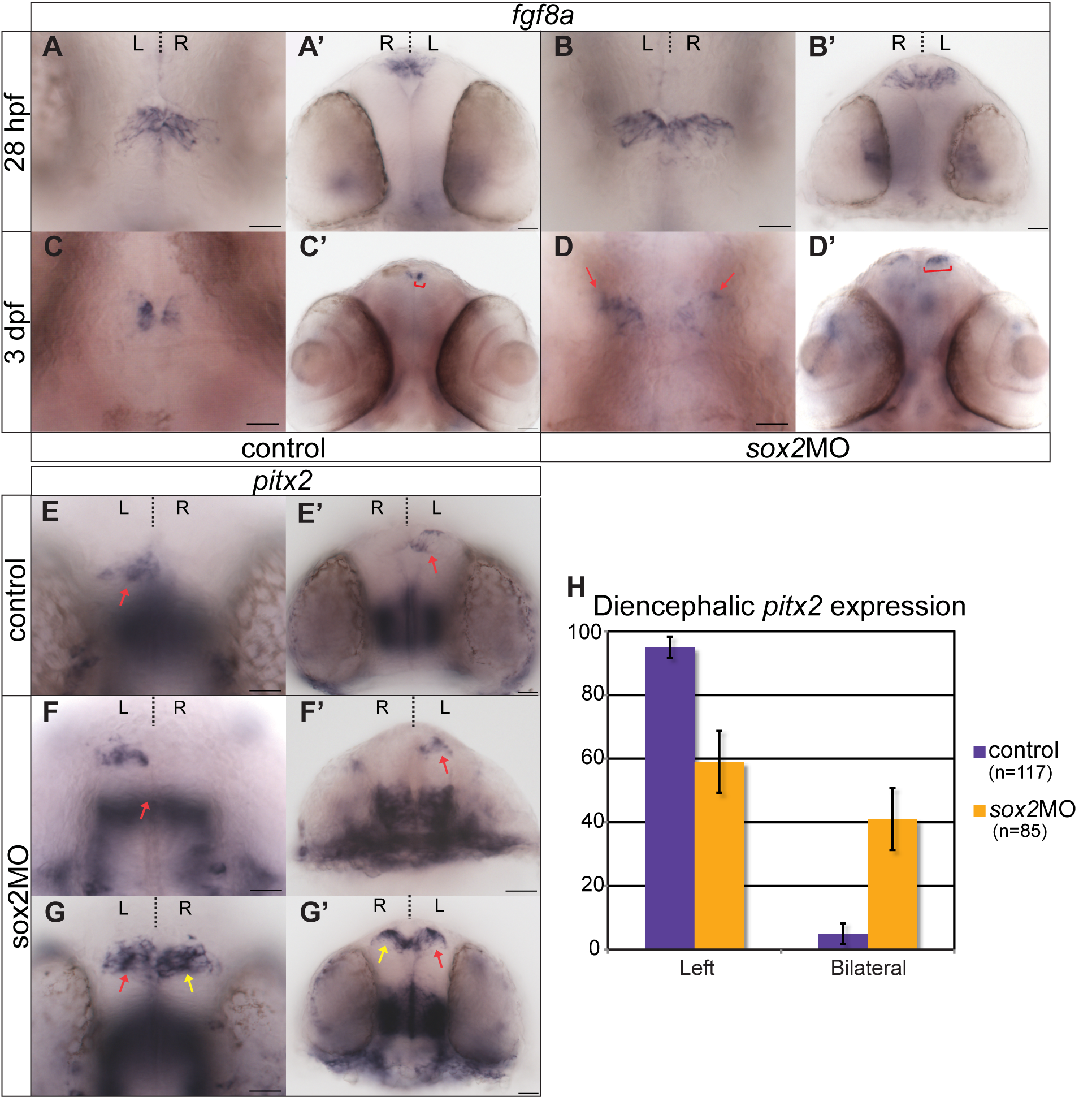

Supplement: Figure S11 — fgf8a and Nodal activity are disrupted in sox2 morphants. (A–D’) fgf8a is normally expressed bilaterally (although higher expression is detected on the left than the right) in the epithalamus, in control embryos (A–A’). As development proceeds, fgf8a expression becomes restricted to the medial part of the diencephalon (C–C’). At early stages, fgf8a expression is normal, in sox2 morphants (B–B’). However, at 3 dpf, fgf8a-positive cells are found in a broader domain when compared to control siblings (red brackets and arrows) (C–D’). (E–E’) pitx2 is normally expressed in the left side of zebrafish diencephalon (red arrow). (F–F’) Approximately 60% of sox2 morphants have normal left-sided pitx2 expression, whereas (G–G’) 40% of embryos have abnormal bilateral pitx2 expression (yellow arrows show abnormal right-sided expression). (H) Average percentage of embryos with left or bilateral pitx2 expression in controls (purple bars) and sox2 morphants (orange bars). (A–G) Dorsal views, (A’–G’) frontal views of the same embryos. Developmental stages are shown at the bottom of each column, scale bars = 25 µm, error bars represent ± standard error. See also Figure 7 – 8 and Movie S4. (TIF) [file pone.0087546.s011.tif]
